# Dietary Mannan Oligosaccharides: Counteracting the Side Effects of Soybean Meal Oil Inclusion on European Sea Bass (*Dicentrarchus labrax*) Gut Health and Skin Mucosa Mucus Production?

**DOI:** 10.3389/fimmu.2015.00397

**Published:** 2015-08-05

**Authors:** Silvia Torrecillas, Daniel Montero, Maria José Caballero, Karin A. Pittman, Marco Custódio, Aurora Campo, John Sweetman, Marisol Izquierdo

**Affiliations:** ^1^Grupo de Investigación en Acuicultura (GIA), Universidad de Las Palmas de Gran Canaria, Las Palmas de Gran Canaria, Spain; ^2^Department of Biology, University of Bergen, Bergen, Norway; ^3^Alltech Aqua, Cephalonia, Greece

**Keywords:** European sea bass, mannan oligosaccharides, soybean oil, GALT, mucus production

## Abstract

The main objective of this study was to assess the effects of 4 g kg^−1^ dietary mannan oligosaccharides (MOS) inclusion in soybean oil (SBO)- and fish oil (FO)-based diets on the gut health and skin mucosa mucus production of European sea bass juveniles after 8 weeks of feeding. Dietary MOS, regardless of the oil source, promoted growth. The intestinal somatic index was not affected, however dietary SBO reduced the intestinal fold length, while dietary MOS increased it. The dietary oil source fed produced changes on the posterior intestine fatty acid profiles irrespective of MOS dietary supplementation. SBO down-regulated the gene expression of *TCR*β*, COX2, IL-1*β*, TNF*α*, IL-8, IL-6, IL-10, TGF*β, and Ig and up-regulated *MHCII*. MOS supplementation up-regulated the expression of *MHCI, CD4, COX2, TNF*α, and *Ig* when included in FO-based diets. However, there was a minor up-regulating effect on these genes when MOS was supplemented in the SBO-based diet. Both dietary oil sources and MOS affected mean mucous cell areas within the posterior gut, however the addition of MOS to a SBO diet increased the mucous cell size over the values shown in FO fed fish. Dietary SBO also trends to reduce mucous cell density in the anterior gut relative to FO, suggesting a lower overall mucosal secretion. There are no effects of dietary oil or MOS in the skin mucosal patterns. Complete replacement of FO by SBO, modified the gut fatty acid profile, altered posterior gut-associated immune system (GALT)-related gene expression and gut mucous cells patterns, induced shorter intestinal folds and tended to reduce European sea bass growth. However, when combined with MOS, the harmful effects of SBO appear to be partially balanced by moderating the down-regulation of certain GALT-related genes involved in the functioning of gut mucous barrier and increasing posterior gut mucous cell diffusion rates, thus helping to preserve immune homeostasis. This denotes the importance of a balanced dietary n–3/n–6 ratio for an appropriate GALT-immune response against MOS in European sea bass juveniles.

## Introduction

The continuous expansion of the aquaculture sector requires securing access to sustainable feed ingredients for intensively cultured fish feed production (1). Over the last 20 years, the aquaculture sector has made a considerable effort to study vegetable oils (VOs) as potential substitutes for fish oil (FO) in aquaculture feeds. However, VOs deficiency in long-chain polyunsaturated fatty acids (LC-PUFAs) and their high 18:C and n–6 fatty acids contents limit their use in aqua feeds, particularly for marine fish species. Marine fish generally have a low Δ6 and Δ5 fatty acyl desaturase activity (2–4) and a low expression of the corresponding genes, despite a certain range of expression modulation through the diet (5), and therefore, their ability to synthesize LC-PUFA from n–6 and n–3 precursors present in VO is very limited. Thus, VO inclusion in marine fish diets reduces the presence of essential fatty acids and alters the n–3/n–6 fatty acids balance and may negatively affect not only fish performance, but also fish health and disease resistance (6).

The negative effect of unbalanced dietary n–3/n–6 fatty acids on marine fish immune competence may depend on the VO fatty acid profile, the VO dietary inclusion levels and the specific ability of the fish species to synthesize LC-PUFA (6). Dietary inclusion of VOs changes the fatty acid composition of cell membranes (6–10), affecting cell function through the modification of the membrane bound enzymes and receptors activities (10, 11). Thus, dietary VOs have been associated with fluctuations on the immune cellular population proportions (10), altered intracellular and extracellular leukocytes killing capacity (9, 10, 12–19), variations in humoral response (9, 12, 15, 17, 18), variations in several physiological processes such as eicosanoids production (4, 9, 20–27), and modulation of signaling molecules and regulators of gene expression (6, 18, 26, 28). Specifically, dietary VO causes several changes in fish that could favor intestinal dysfunction, such as variations in tissue composition and morphology (19, 29–34), intestinal functionality (6, 35–38), variations in microbiota profiles (39, 40), or alterations in the gut-associated immune system (GALT) function (6). In addition, dietary alterations could stimulate the rate of production of mucous (41–43) and its biochemical composition (44), thus regulating the intestinal dynamic barrier formed by epithelium cells (45).

One of the possible strategies to counteract the side effects of high dietary VO on fish health, other than balancing the n–3/n–6 ratio with VO blends, could be to incorporate natural feed additives, such as mannan oligosaccharides (MOS), that would help to maintain tissue structure and promote the innate immune system. Dietary MOS has been demonstrated to promote fish performance and disease resistance by stimulating the systemic and GALT systems and by reinforcing the gut epithelial barrier structure and functionality in FO-based diets (44).

Thus, the main objective of this study is to assess the effects of dietary MOS supplementation in soybean oil (SBO)- and FO-based diets on the gut health of European sea bass (*Dicentrarchus labrax*) juveniles in relation to gut morphology and functionality.

## Materials and Methods

### Diets

Diets were designed to contain FO or SBO as the single lipid source in combination with 4 g ⋅ kg^−1^ MOS (Biomos^®^; Alltech, Inc., USA) replacing standard carbohydrates (corn meal) (FOMOS and SBOMOS). Extruded diets (2–3 mm slow sinking pellets; 6–8% moisture) were isolipidic and isoproteic and manufactured by Sparos, Lda (CRIA-Universidade do Algarve, Faro, Portugal). Dietary formulation, proximate composition, and fatty acid profiles are indicated in Tables 1 and 2.

**Table 1:** **Main ingredients and composition of the experimental diets**.

Ingredient	Diet (g ⋅ kg^−1^ dry weight)			
	FO	FOMOS	SBO	SBOMOS
Fish meal^a^	515.0	515.0	515.0	515.0
Soybean meal^b^	97.8	97.8	97.8	97.8
Wheat	85.3	85.3	85.3	85.3
Wheat gluten	85.3	85.3	85.3	85.3
Corn meal^c^	65.3	61.3	65.3	61.3
Fish oil^d^	147.2	147.2	0	0
Soybean oil	0	0	147.2	147.2
Mineral and vitamin mix^e^	4	4	4	4
Antioxidant (BHT)	0.1	0.1	0.1	0.1
MOS^f^	0	4	0	4
**Composition (%, dry weight)**				
Crude lipids	21.12	21.55	21.23	21.23
Crude protein	48.18	48.77	47.71	47.43
Moisture	4.29	4.73	3.91	3.90
Ash	10.55	10.42	11.46	11.41

*^a^Peruvian fish meal (67% crude protein; 9% crude fat; EXALMAR, Peru)*.

*^b^Soybean meal (Solvent extracted dehulled soybean meal; 47% crude protein; 2.6% crude fat; SORGAL SA, Portugal)*.

*^c^Corn meal (8–12% crude protein)*.

*^d^Peruvian fish oil*.

*^e^Mineral and vitamin premix provided by BioMar SAS*.

*^f^Biomos^®^, Alltech, Inc., USA*.

**Table 2:** **Main fatty acids composition (% of total identified fatty acids) of experimental diets**.

	FO	FOMOS	SBO	SBOMOS
Saturated	11.72 ± 0.06	11.75 ± 0.02	5.72 ± 0.02	5.75 ± 0.02
Monoens	25.81 ± 0.37	25.89 ± 0.04	27.08 ± 2.16	24.57 ± 1.18
n–3	34.00 ± 0.10	34.35 ± 0.01	9.72 ± 1.10	10.87 ± 1.32
n–6	7.06 ± 0.21	6.72 ± 0.04	42.86 ± 1.14	44.25 ± 0.25
n–9	9.97 ± 0.04	9.78 ± 0.02	22.20 ± 1.63	19.91 ± 0.14
n–3 LC-PUFA	28.63 ± 0.08	28.96 ± 0.05	4.35 ± 0.93	5.28 ± 1.38
n–3/n–6	4.82 ± 0.13	5.11 ± 0.03	0.24 ± 0.02	0.25 ± 0.03
20:4n–6	1.23 ± 0.11	1.21 ± 0.02	0.19 ± 0.09	0.27 ± 0.14
20:5n–3	15.09 ± 0.09	15.35 ± 0.06	1.61 ± 0.17	1.81 ± 0.29
22:6n–3	10.74 ± 0.03	10.81 ± 0.11	2.31 ± 0.72	3.01 ± 1.00

### Experimental conditions

Prior to the feeding trial, European sea bass juveniles (960 fish) were maintained in stocking tanks and fed a commercial extruded diet for three weeks. Fish were then randomly distributed in 12 indoor cylindroconical 1000 L fiberglass tanks at an initial stocking density of 1.7 kg ⋅ m^−3^ (80 fish/tank). Fish average initial weight and length were 20.63 ± 0.12 g and 11.73 ± 0.07 cm, respectively (mean ± SD). Tanks were supplied with filtered sea water at a temperature of 19.0–20.3°C in a flow-through system and natural photoperiod (12L:12D). Dissolved oxygen oscillated between 7.3 and 8.1 ppm. Fish were manually fed until apparent satiation with one of the four experimental diets for 60 days (three times a day, 6 days a week). Each dietary treatment was assayed in triplicate.

Sampling was performed after 60 days of feeding. All fish were sampled for final weight and length after 24 h of starvation. Ten fish per tank were sampled individually for skin and gut histological studies. Additionally, posterior gut of six fish per tank was taken and quickly kept in RNA later and frozen at −80°C until gene expression analysis. Five fish per tank were sampled for intestinal somatic index (ISI), after 24 h of starvation, and pooled for posterior gut proximate composition and fatty acid analyses. The animal experiments described comply with the guidelines of the European Union Council (2010/63/EU) for the use of experimental animals and have been approved by the Bioethical Committee of the University of Las Palmas de Gran Canaria.

### Biochemical analyses

Feed and tissue biochemical composition analyses were carried out using standard procedures (46). Crude protein content (Nx6.25) was determined by the Kjeldahl method and crude lipid was analyzed as described in Ref. (47). Moisture was determined by thermal dehydration to constant weight at 110°C. Ash content was determined by combustion at 600°C for 12 h. Fatty acid methyl esters profiles were prepared by transmethylation (48) and separated by gas chromatography (GC-14A, Shimadzu, Japan) following the conditions described in reference (49). Fatty acid methyl esters quantification was performed using a flame ionizator detector and identification by comparison with external and well-characterized FOs standards (EPA 28, Nippai, Ltd., Tokyo, Japan). All analyses were conducted in triplicate.

### Histological studies

#### Gut Morphometric Analyses

Fish intestines were dissected out and six to eight transversal sections of the posterior intestine (from the first diffused sphincter to rectum sphincter; *N* = 15 per group) were fixed in 4% neutral-buffered formalin and embedded in paraffin perpendicularly to the bottom of the mold. For each specimen, six serial sections (5 μm thick) were stained with H&E and/or specific Alcian Blue/PAS staining (pH = 2.5) (50). Micrographs were taken at a final magnification of 200× and 400× using a Nikon Microphot-FXA microscope and an Olympus DP50 camera (*n* = 5 × 5 per individual fish; 125 images per feeding group). Mucosal fold height and width were determined using an analySIS^®^ (Image Pro Plus^®^, Media Cybernetics, Silver Spring, MD, USA) software package (51).

#### Mucosal Mapping

Sections of skin (from the dorsolateral area just below the dorsal fin), anterior, and posterior gut (five fish/tank; *N* = 15 groups) were removed at necropsy and fixed in buffered 4% formalin. The tissues were dehydrated in a series of ethanol dilutions and embedded progressively in Technovit (Electron Microscopy Sciences, PA, USA). Histological sections of 3 μm thickness were stained with PAS-Alcian Blue for unequivocal marking of mucous cells. The stereological analysis was performed from live images of sections with Visiopharm v.3.0 software (Visiopharm, Hoersholm, Denmark). Mucosal Mapping [see Ref. (52) for details] was applied to the intestinal and skin mucosal tissues to evaluate responses in the innate immune system. Five unbiased measures were obtained: epithelial tissue area (μm^2^), number of mucous cells, mucus cell area (μm^2^) with a correction factor for plane of sectioning (53), mucous cell density in the epithelium (%), and ratio of epithelium to mucus cell (E:M).

### RNA extraction and real-time quantitative PCR analysis

Total RNA of the fish posterior intestine (*N* = 2 pools of three fish/tank; *N* = 6/diet) was extracted using TRI reagent (Sigma-Aldrich, Saint Louis, MO, USA) and quantified by spectrophotometry (Nanodrop 1000, Themo Fisher Scientific Inc., USA). The integrity of RNA was verified by Gel Red™ staining (Biotium Inc., Hayward, CA, USA) on a 1.4% agarose gel. The reverse transcription (RT) reactions were performed using the i*Script*™cDNA Synthesis Kit (Bio-Rad Hercules, California) in 20-μL reaction volume containing 1 μg of total RNA.

Fifteen genes involved in the immune system response (*IL-1*β*, IL-10, TNF*α*, IL-8, IL-6, CD4, CD8*α*, MHCI, MHCII, TCR*β*, TGF*β*, COX2, Ig, Caspase 3*, and *Caspase 9*) were analyzed by real-time PCR, using β*-actin* as housekeeping gene. The PCRs were carried out in a*I-cycler* with optical module (Bio-Rad Hercules, CA, USA) in a final volume of 20 μL containing 10 μL of Brillant SYBR Green QPCR Master Mix (Bio-Rad Hercules, CA, USA), 0.6 μL of each primer (10 mM), and 2 μL of cDNA (1:10 dilution). The PCR conditions for genes *CD4, CD8*α*, MHCI, MHCII, TCR*β*, TGF*β*, COX2, IL-10*, and *Ig* were as follows: 95°C for 10 min, followed by 35 cycles of 95°C for 45 s, 52°C for 45 s and 72°C for 45 s, as described before (54). Reaction mixtures for genes *IL-8, IL-6, Caspase 3*, and *9* were incubated for 10 min at 95°C, followed by 35 cycles of 45 s at 95°C, 45 s at 60°C, and 72°C for 45 s. The cycling conditions for *IL-1*β and *TNF*α were 95°C for 10 min, followed by 40 cycles of 95°C for 15 s, 58°C for 30 s, and 72°C for 30 s. Each run ended with a melting curve analysis resulting in a melting peak profile specific for the amplified target DNA. Reactions were performed in duplicate for each template cDNA. Blank control reactions replaced cDNA with water. Relative gene expression was estimated by the Δ–Δ method (55). The specific primers used are indicated in Table 3.

**Table 3:** **Primers used for gene expression analysis by real-time PCR in posterior gut of European sea bass (*Dicentrarchus labrax*) fed cMOS (*t* = 60 days)**.

Gene	Name	Accession number	Forward (5′ → 3′)	Reverse (5′ → 3′)
*IL-1*β	Interleukin 1β	AJ269472	ATTACCCACCACCCACTGAC	TCTCTTCCACTATGCTCTCCAG-
*IL-10*	Interleukin 10	AM268529	ACCCCGTTCGCTTGCCA	CATCTGGTGACATCACTC
*TNF*α	Tumor necrosis factor α	DQ200910	ACAGCGGATATGGACGGTG	GCCAAGCAAACAGCAGGAC
*IL-8*	Interleukin 8	AM490063	GTCTGAGAAGCCTGGGAGTG	GCAATGGGAGTTAGCAGGAA
*IL-6*	Interleukin 6	AM490062	ACTTCCAAAACATGCCCTGA	CCGCTGGTCAGTCTAAGGAG
*CD4*	T-cell surface glycoprotein CD4	AM849811	GTGATAACGCTGAAGATCGAGCC	GAGGTGTGTCATCTTCCGTTG
*CD8*α	T-cell surface glycoprotein CD8 α chain	AJ846849	CTAAGATTCGGCAAAATAACTCGAC	GATGAGGAGTAGAAGAAGAAGGCC
*MHCI*	Major histocompatibility complex class I	AM943118	CAATACCTCACCCAGA	CTCCATCTTTCCTCCAGAT
*MHCII*	Major histocompatibility complex class II	AM113466	CAGAGACGGACAGGAAG	CAAGATCAGACCCAGGA
*TCR*β	T-cell antigen receptor β chain	AJ493441	GACGGACGAAGCTGCCCA	TGGCAGCCTGTGTGATCTTCA
*TGF*β	Transforming growth factor β	AM421619	GACCTGGGATGGAAGTGG	CAGCTGCTCCACCTTGTG
*COX2*	Ciclo-oxigenase 2	AJ630649	CATTCTTTGCCCAGCACTTCACC	AGCTTGCCATCCTTGAAGAGTC
*IgL#18 (Ig)*	Immunoglobulin	AJ400233	GAGCTGCAGGAGGACAGTG	TCAGACTGGCCTCACAGCT
*Casp-3*	Caspase 3	DQ345773	CTGATTTGGATCCAGGCATT	CGGTCGTAGTGTTCCTCCAT
*Casp-9*	Caspase 9	DQ345775	GGCAGGACTCGACGAGATAG	CTCGCTCTGAGGAGCAAACT
β*-actin*	β-actin	AJ537421	ATGTGGATCAGCAAGCAGG	AGAAATGTGTGGTGTGGTCG

### Statistical analyses

All data were tested for normality and homogeneity of variance. Means and SDs were calculated for each parameter measured. When required, data arcsine square root transformation was performed, particularly when data were expressed as % (56). Statistical analyses followed methods outlined in ref. (57). The effects of oil source (SBO and FO) and MOS supplementation (0 or 4 g ⋅ kg^−1^) were analyzed by two-way ANOVA, where oil source or MOS supplementation were established as fixed factors. Significant differences were considered when *P* < 0.05. Linear models (lme) were used to correlate individual genes with measures of mucous cell area, density, and their ratio. Further redundancy analysis (RDA) was performed to correlate the immune gene expression with the histological structures configuration. Analyses were performed using the SPSS Statistical Software System v20.0 (SPSS, Chicago, IL, USA) and R (version 3.1.0).

## Results

### Growth performance and intestinal somatic index

Whereas dietary inclusion of MOS significantly increased fish SGR as denoted by the two-way ANOVA [*F*(1,8) = 5.651, *P* < 0.05], complete replacement of FO by SBO only showed a tendency to reduce fish SGR (Table 4). Final fish weight (g ± SD) ranged between 38.06 ± 0.15 for fish fed SBOMOS and 39.97 ± 1.32 for fish fed FOMOS (58). No interaction was found between dietary oil (O) and MOS supplementation for SGR or an effect of MOS or SBO inclusion on ISI (Table 4).

**Table 4:** **Growth performance and intestinal somatic index of European sea bass (*Dicentrarchus labrax*) fed the experimental diets at the end of the feeding trial (*t* = 60 days)**.

	SGR^a^	ISI (%)^b^
FO	1.10 ± 0.07	2.33 ± 0.19
FOMOS	1.19 ± 0.04	2.37 ± 0.08
SBO	1.08 ± 0.01	2.21 ± 0.13
SBOMOS	1.10 ± 0.02	2.19 ± 0.19
**Two-way anova**		
Dietary oil (O)	NS	NS
MOS	*P* < 0.05	NS
O × MOS	NS	NS

*Values expressed in mean ± SD (*n* = 3 tanks/diet). Different letters within a column denote significant differences (*P* < 0.05) based on Tukey’s multiple range test (one-way ANOVA)*.

*^a^Specific growth rate (SGR) = [(ln final weight – ln initial weight)/number of days] × 100*.

*^b^Intestinal index (ISI) = (wet intestine weight/wet body weight) × 100*.

*NS, not significant*.

### Posterior gut lipid content and fatty acid profiles

Dietary MOS, oil source, or their interaction did not affect the posterior intestine lipid content. Lipid percentages ranged from 27.99 ± 1.55 in fish fed SBOMOS to 30.48 ± 1.54 in fish fed FOMOS (g ⋅ 100 g^−1^ dry weight). Substitution of dietary FO by SBO resulted in lower (*P* < 0.05) saturated, n–3, n–3LC-PUFA, and n–3/n–6 fatty acids and higher (*P* < 0.05) n–6 and n–9 fatty acids contents in posterior intestine (*P* < 0.05), regardless of dietary MOS inclusion. Indeed, the two-factor analysis of variance mainly attributed the variation in the main classes of fatty acids in tissue (*P* < 0.05) to dietary oil source irrespective of MOS dietary supplementation (Table 5). Thus, fish fed SBO diets presented a 8% lower posterior gut saturated fatty acids content when compared to fish fed FO-based diets, despite the 52% lower dietary content. This reduced the dietary/tissue content ratio from 0.95 in fish fed FO-based diets to 0.5 in fish fed SBO diets. Similarly, despite the 70% and 83% lower content of n–3 and n–3LC-PUFA in SBO-based diets, tissue percentages were reduced only by 37% and 43%, respectively. The DHA posterior intestine content was only reduced in fish fed SBO diet in relation to the rest of the treatments and, despite the 80% lower dietary ARA content for fish fed SBO-based diets, fish fed FOMOS, SBO, and SBOMOS diets presented almost similar posterior gut percentages, denoting a selective retention in this intestinal region. Indeed, the dietary/tissue ARA percentage ratio oscillated from 0.72 in fish fed FO-based diets to 0.18 for fish fed SBO-based diets (Table 5). Only the saturated fatty acids were significantly reduced by dietary MOS inclusion (*F*(1,8) = 6.31; *P* < 0.05) (Table 5). There was no interaction between oil source and prebiotic supplementation (Table 5).

**Table 5:** **Posterior intestine main classes fatty acids profile (% of total identified fatty acids) of European sea bass (*Dicentrarchus labrax*) fed the experimental diets at the end of the feeding trial (*t* = 60 days)**.

	Posterior Intestine						
	FO	FOMOS	SBO	SBOMOS	MOS	OIL	M × O
Saturated	12.31 ± 0.36	12.21 ± 0.09	11.56 ± 0.16	11.08 ± 0.04	*P* < 0.05	*P* < 0.05	NS
Monoens	28.97 ± 2.53	29.99 ± 1.10	27.14 ± 0.36	27.77 ± 0.27	NS	*P* < 0.05	NS
n–3	27.73 ± 2.97	27.74 ± 3.09	16.67 ± 1.92	17.86 ± 0.18	NS	*P* < 0.05	NS
n–6	10.92 ± 0.32	10.65 ± 0.83	27.70 ± 1.47	27.19 ± 0.82	NS	*P* < 0.05	NS
n–9	17.35 ± 1.33	18.02 ± 1.03	20.47 ± 0.28	20.86 ± 0.11	NS	*P* < 0.05	NS
n–3 LC-PUFA	24.81 ± 3.17	24.94 ± 3.12	13.54 ± 2.04	14.54 ± 0.15	NS	*P* < 0.05	NS
n–3/n–6	2.54 ± 0.23	2.63 ± 0.50	0.60 ± 0.09	0.66 ± 0.03	NS	*P* < 0.05	NS
20:4n–6	1.74 ± 0.37	1.65 ± 0.10	1.33 ± 0.12	1.24 ± 0.03	NS	*P* < 0.05	NS
20:5n–3	9.45 ± 0.59	9.67 ± 1.04	3.60 ± 0.82	3.89 ± 0.34	NS	*P* < 0.05	NS
22:6n–3	13.26 ± 2.54	13.05 ± 1.85	8.89 ± 1.09	9.42 ± 0.22	NS	*P* < 0.05	NS

*Different letters within a line denote significant differences (*P* < 0.05) among dietary treatments (one-way ANOVA). Values expressed in mean ± SD (*n* = 3 tanks/diet)*.

*NS, not significant*.

### Morphological studies

#### Posterior Intestine Morphometry

**Figure 1:**
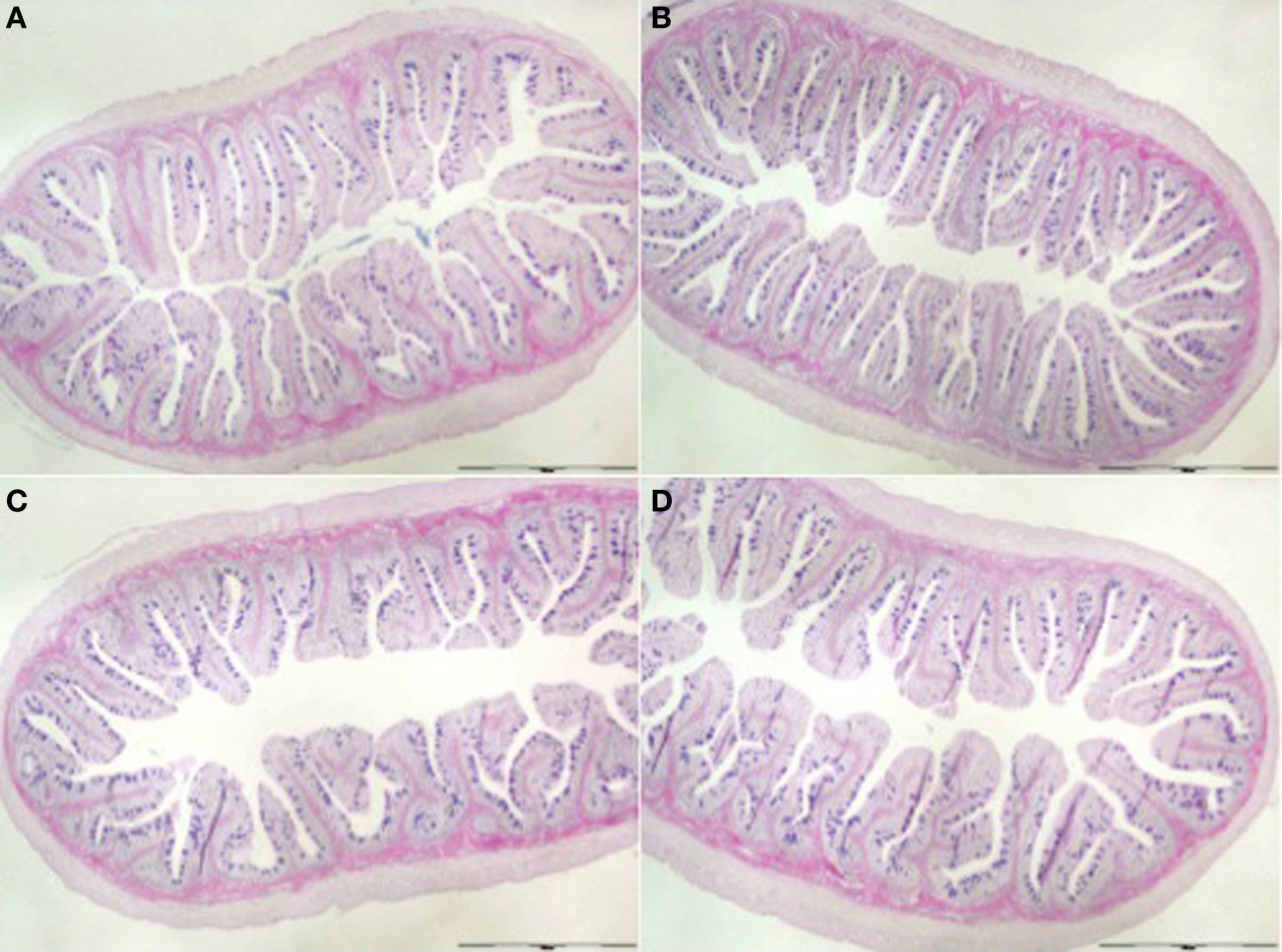
**Posterior gut (Alcian Blue-PAS) from fish fed (A) Fish oil-based diet (FO), (B) FO- and MOS-based diet (FOMOS), (C) soy bean meal-based diet (SBO), and (D) SBO- and MOS-based diet (SBOMOS) after 60 days of supplementation**.

Morphological evaluation of the posterior gut sections revealed an intact epithelial barrier, organized villi, lack of debris in the lumen and no signs of edema or vasodilatation for all dietary treatments (Figure 1). Two-way ANOVA analyses revealed the effect on fold length of dietary oil source [*F*(1,8) = 96.25, *P* < 0.05] and MOS [*F*(1,8) = 52.57, *P* < 0.05], as well as the interaction between both factors [*F*(1,8) = 23.31, *P* < 0.05] (Table 6; Figure 1). Indeed, fish fed FOMOS diet had the longest intestinal folds and fish fed SBO diet had the shortest when compared with the rest of the groups. There was no effect (*P*>0.05, *n* = 250/tank) of MOS or dietary oil source or their combination on the mucosal folds width, although a trend to wider folds was observed in fish fed FOMOS diet (Table 6).

**Table 6:** **Folding intestine patterns for European sea bass (*Dicentrarchus labrax*) posterior intestine after 8 weeks of feeding trial**.

	Dietary treatment						
	FO	FOMOS	SBO	SBOMOS	MOS	OIL	M × O
Length (μm)	183.72 ± 3.50	186.90 ± 1.06	164.52 ± 1.32	180.36 ± 2.35	*P* < 0.05	*P* < 0.05	*P* < 0.05
Width (μm)	45.98 ± 1.44	50.82 ± 1.20	46.59 ± 4.99	46.95 ± 4.03	NS	NS	NS

*Different letters within a line denote significant differences (*P* < 0.05) among dietary treatments. Values expressed in mean ± SD (*n* = 3 tanks/diet; *n* = 5 fish/tank)*.

*NS, not significant*.

#### Mucosal Mapping™

**Figure 2:**
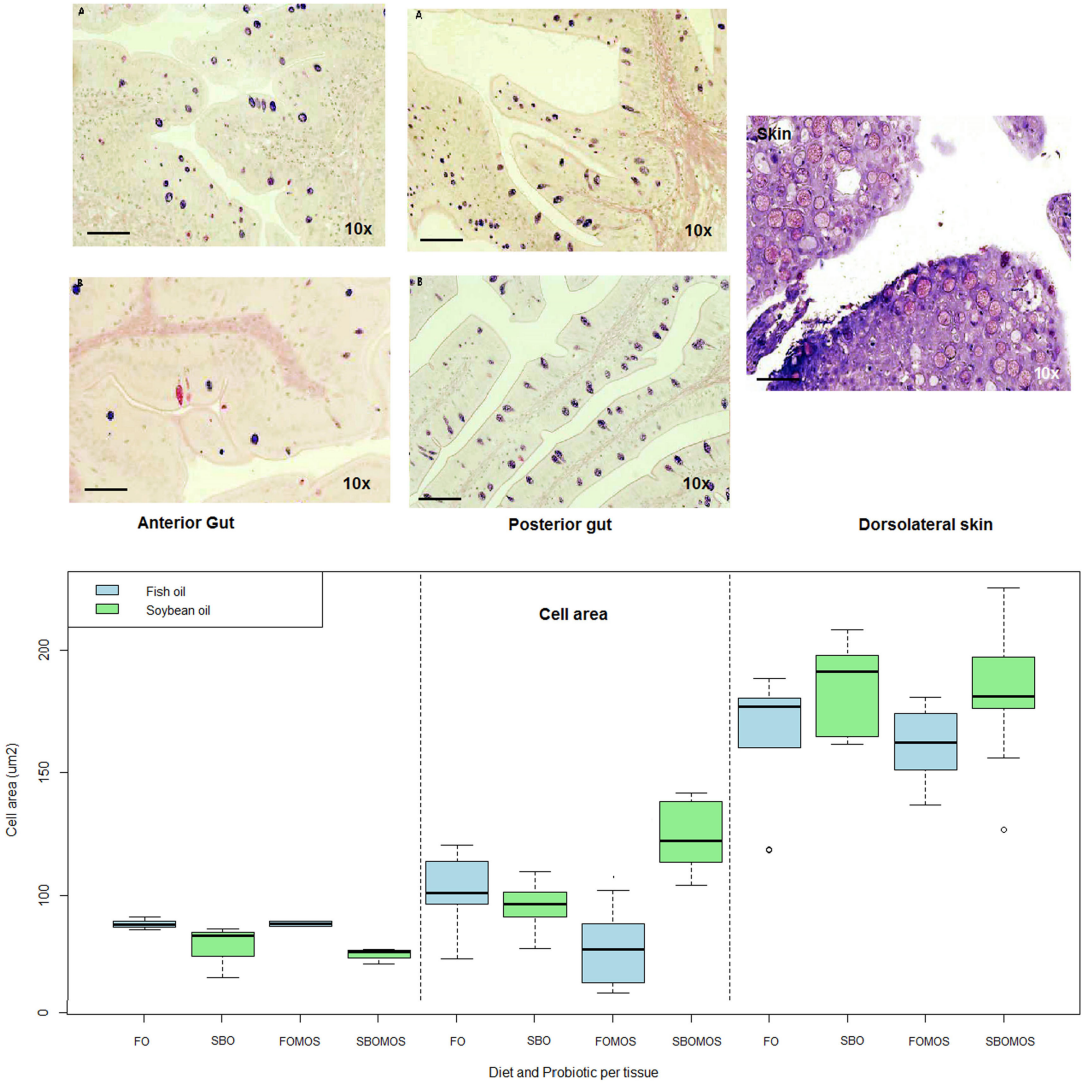
**Top left: Histological sections of anterior gut (left) and posterior gut (center) fed with (A) FO- and (B) SBO-based diets, stained in Alcian Blue-PAS**. Top right: histological section of skin fed with fish oil and stained with Toluidine Blue (scale bar = 300 μm). Bottom: Mucous cell area (μm^2^) depending on tissue and diet. From left to right: anterior gut, posterior gut, and skin, differentiated significantly by a dotted line (*P* = 2e–16, *F*-test). Green color is soybean oil and blue color is fish oil. *N* = 36 fishes.

In general, grand mean mucous cell areas patterns differed (*P* < 0.05) among tissues, with the largest mucous cells in the skin (170 μm^2^), followed by posterior gut (90 μm^2^), and the smallest in the anterior gut with an average of 82 μm^2^ (Figure 2). Additionally, there were significant differences in mean mucous cell areas within the posterior gut where both SBO and dietary MOS induced smaller cell areas (*P* < 0.05). Nevertheless, the two dietary components interact in a manner such that the addition of MOS to a SBO-based diet increases the mucous cell size over the values shown in fish fed FO-based diet. Finally in the skin and anterior gut, no differences were found due to dietary oil source or MOS supplementation.

**Figure 3:**
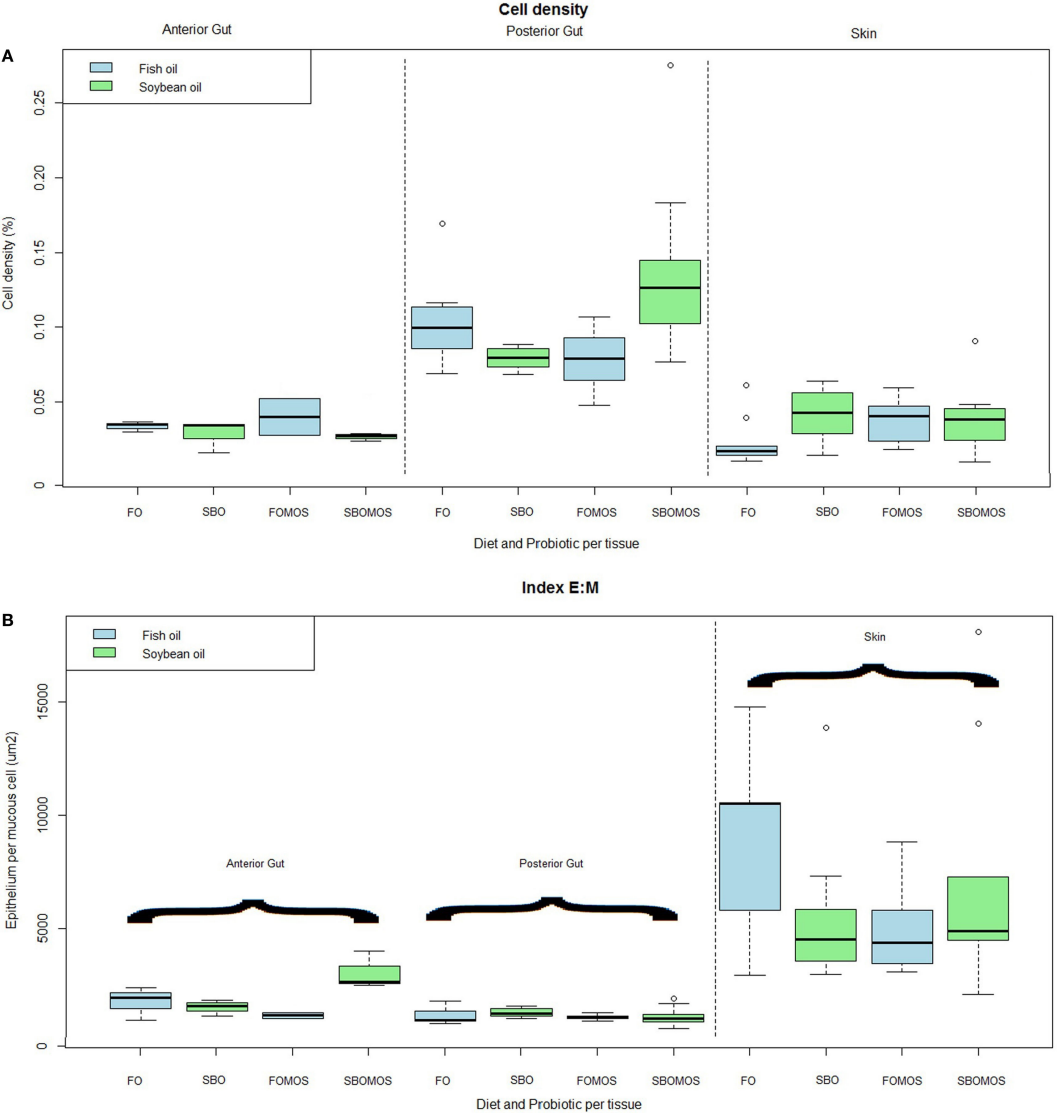
**(A)** Cell density (%) depending on the tissue and diet. From left to right: anterior gut, posterior gut, and skin, differentiated significantly by a dotted line (*P* = 4.23e–14, *F*-test). Green color is SBO and blue FO. *N* = 36 fishes. **(B)** Total epithelium per mucous cell (μm^2^) depending on the tissue and the diet. Organs are significantly separated by a dotted line (*P* = 1.2e–11, *F*-test) being anterior and posterior gut in the left side, and skin in the right side of the line. Green color is soybean oil and blue fish oil. *N* = 36 fishes.

The overall density of mucous cells in the epithelia was also affected by tissue. The posterior gut had a significantly higher density of mucous cells (*P* < 0.05), with about 10% of the mucosal epithelium composed of mucous cells compared to about 3.6% in the dorsolateral skin and 3.8% in the anterior gut. Within these tissues, the anterior gut shows a trend (*P* = 0.05) to lower mucous cell density in fish fed SBO than in fish fed FO, regardless of dietary MOS inclusion (Figure 3A).

The ratio of epithelium to mucous cells (E:M) also shows tissue differences. The mean E:M ratio in both sections of the intestine was relatively low (between about 1500 and 3000 μm^2^ of epithelium per mucous cell) which is lower (*P* < 0.05) than that of the dorsolateral skin (Figure 3B). The SE of the mucous cell area measurements remains below the 10%, while all the other indexes remain below 30% (data not shown).

### Posterior gut gene expression studies

No significant differences were observed in the expression of caspase-encoding genes among fish fed the different dietary treatments (data not shown). However, after 60 days of feeding, dietary treatments significantly affected the relative expression of *MHCII, MHCI, CD4, CD8*α*, TCR*β*, COX2, IL-1*β*, TNF*α*, IL-8, IL-6, TGF*β*, IL-10*, and *Ig* genes in the posterior gut of European sea bass (Table 7). In general, the two-way ANOVA test (Table 7) showed that dietary inclusion of SBO down-regulated (*P* < 0.05) the expression of *TCR*β*, COX2, IL-1*β*, TNF*α*, IL-8, IL-6, IL-10, TGF*β, and Ig genes and up-regulated (*P* < 0.05) *MHCII* gene expression (Table 7). Furthermore, results obtained indicate that MOS dietary inclusion affected (*P* < 0.05) *MHCI, CD4, CD8*α*, COX2, TNF*α*, IL-8, IL-6, TGF*β, and *Ig* transcript levels regardless of dietary SBO supplementation (Table 7). An effect (*P* < 0.05) of the combination of both factors (MOS × oil source) was detected on *MHCI, CD4, COX2, IL-1*β*, IL-6, TGF*β, and *Ig* relative gene expression. Particularly, it seems that inclusion of MOS in the SBO-based diet trends to up-regulate *IL-6, TGF*β, and *IL-10* gene expression and in a lesser extends, *IL-1*β gene expression, counteracting the gene down-regulation effect of dietary SBO in comparison to dietary FO. Besides, the up-regulating effect of MOS inclusion in the FO-based diet on gene expression of *MHCI, CD4, CD8*α, *COX2, TNF*α, and *Ig* seems to be reduced when included in SBO-based diet (Table 7).

**Table 7:** **Posterior gut relative gene expression profile of European sea bass (*Dicentrarchus labrax*) juveniles determined by RT-PCR after 60 days of dietary supplementation**.

	Dietary treatment				Two-way ANOVA		
	
	FO	FOMOS	SBO	SBOMOS	MOS	OIL	M × O
*MHCII*	1.01 ± 0.12	1.14 ± 0.04	2.16 ± 0.43	1.88 ± 0.32	NS	*P* < 0.05	NS
*MHCI*	1.03 ± 0.26	3.13 ± 0.15	0.77 ± 0.12	1.00 ± 0.26	*P* < 0.05	*P* < 0.05	*P* < 0.05
*CD4*	1.10 ± 0.17	2.02 ± 0.28	0.77 ± 0.09	1.07 ± 0.14	*P* < 0.05	NS	*P* < 0.05
*CD8*α	1.04 ± 0.25	1.62 ± 0.46	1.11 ± 0.33	1.49 ± 0.32	*P* < 0.05	*P* < 0.05	NS
*TCR*β	1.04 ± 0.14	1.44 ± 0.14	0.82 ± 0.30	0.81 ± 0.30	NS	*P* < 0.05	NS
*COX2*	1.09 ± 0.16	1.76 ± 0.13	0.50 ± 0.09	0.66 ± 0.09	*P* < 0.05	*P* < 0.05	*P* < 0.05
*IL-1*β	1.06 ± 0.16	1.02 ± 0.08	0.53 ± 0.13	0.83 ± 0.10	NS	*P* < 0.05	*P* < 0.05
*TNF*α	1.04 ± 0.12	1.98 ± 0.26	0.87 ± 0.13	1.27 ± 0.43	*P* < 0.05	*P* < 0.05	NS
*IL-8*	1.08 ± 0.38	0.75 ± 0.11	0.71 ± 0.21	0.35 ± 0.20	*P* < 0.05	*P* < 0.05	NS
*IL-6*	1.05 ± 0.22	0.57 ± 0.01	0.39 ± 0.05	0.48 ± 0.04	*P* < 0.05	*P* < 0.05	*P* < 0.05
*TGF*β	1.06 ± 0.17	0.26 ± 0.02	0.32 ± 0.02	0.60 ± 0.05	*P* < 0.05	*P* < 0.05	*P* < 0.05
*IL-10*	1.02 ± 0.13	1.06^a^ ± 0.06	0.60 ± 0.09	0.75 ± 0.05	NS	*P* < 0.05	NS
*IgL#18*	1.06 ± 0.23	1.75 ± 0.03	0.48 ± 0.14	0.58 ± 0.23	*P* < 0.05	*P* < 0.05	*P* < 0.05

*Different letters within a line denote significant differences (*P* < 0.05) among dietary treatments (one-way ANOVA; Tukey’s test). Values expressed in mean ± SD of two pools of three specimens analyzed per tank (*n* = 3 tanks/diet)*.

*NS, not significant*.

**Figure 4:**
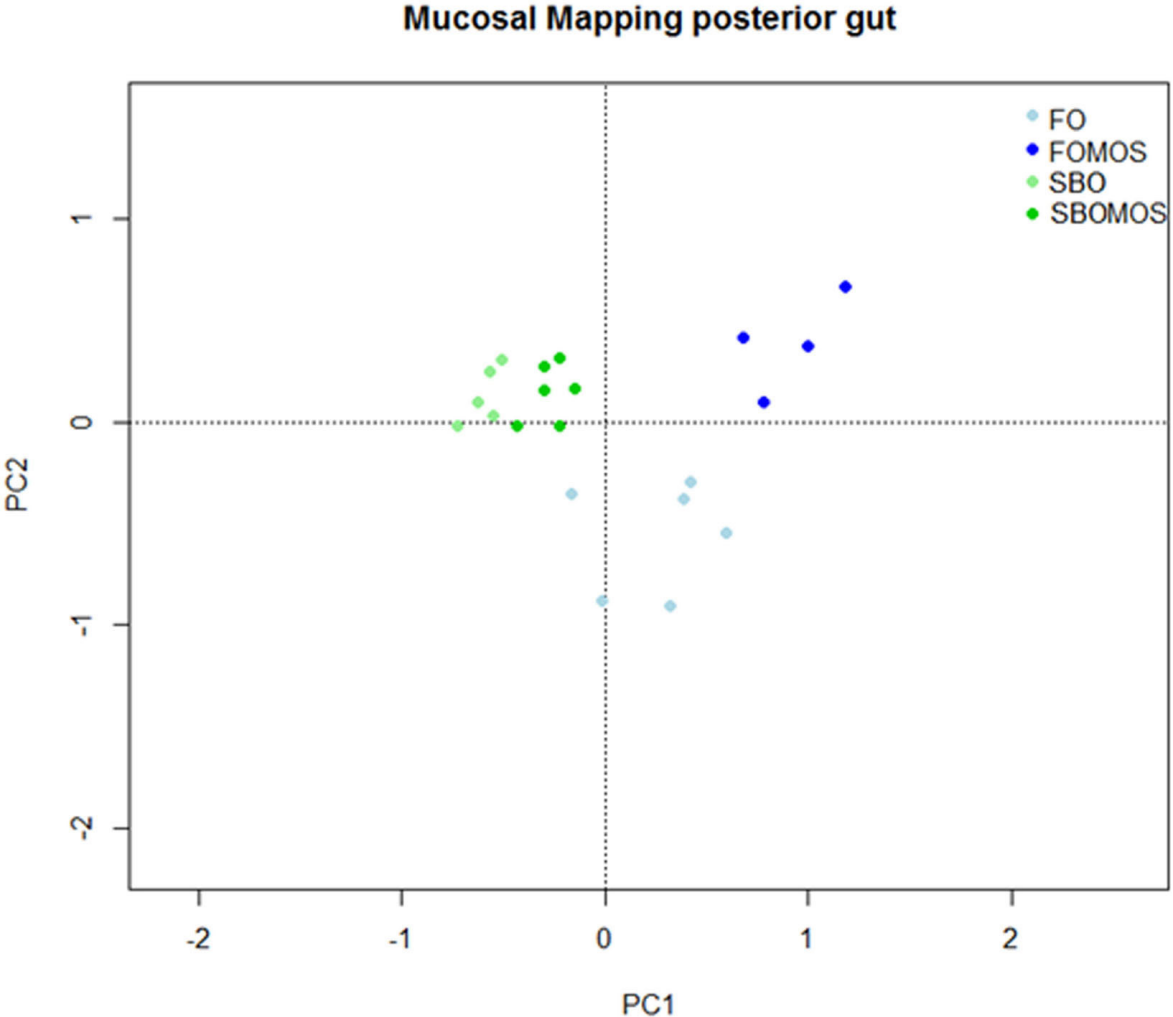
**Principal component analysis (PCA) to study the variability on the posterior gut relative gene expression in relation to dietary treatment**. The points correspond to the tanks and are colored according to their diet, being FO, fish oil; FOMOS, fish oil and MOS; SBO, soybean oil; SBOMOS, soybean oil and MOS.

**Figure 5:**
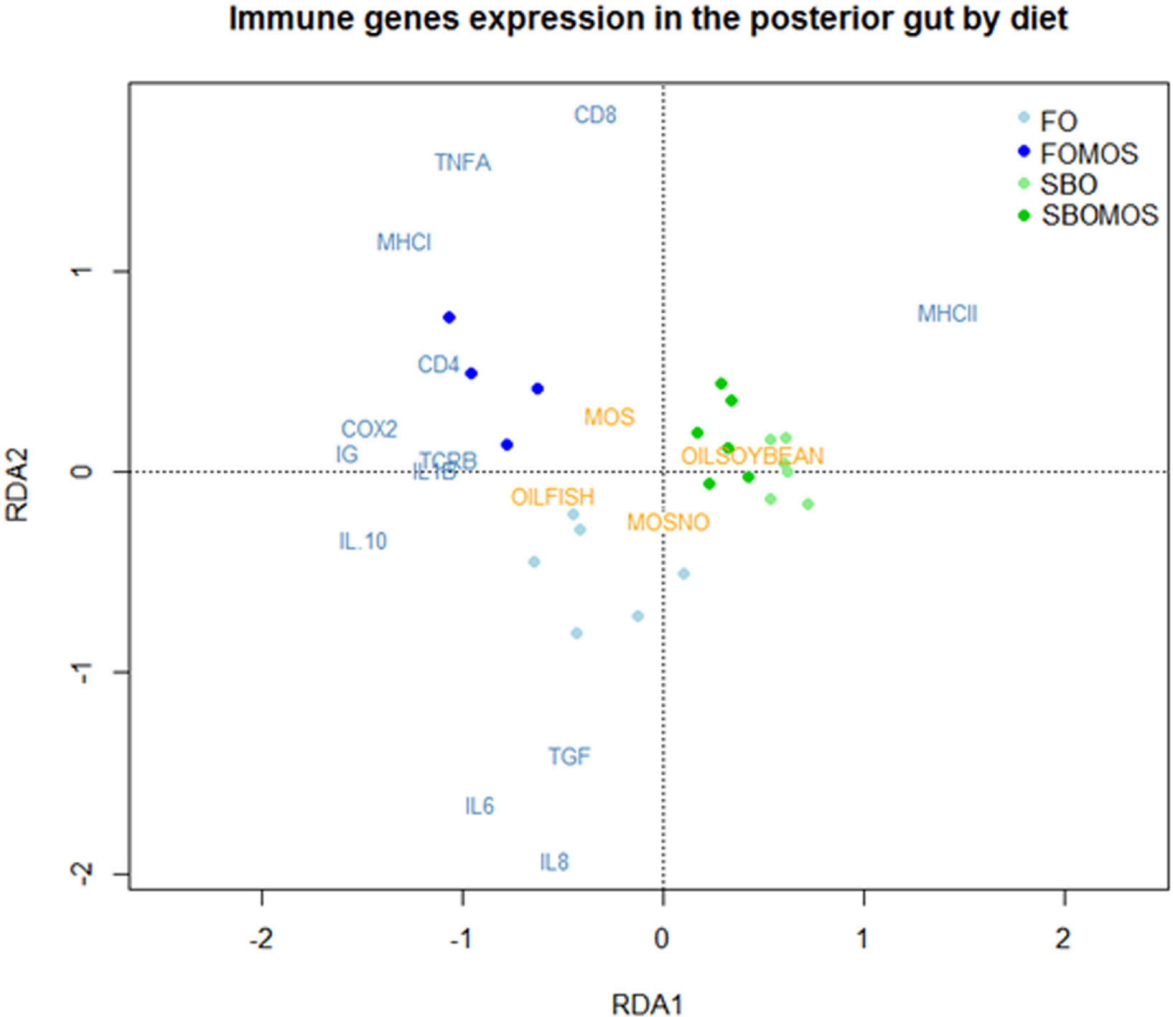
**Redundancy analysis (RDA) to study the variability posterior gut relative gene expression as response dietary treatment**. The points correspond to the tanks and are colored according to their diet, being FO, fish oil; FOMOS, fish oil and MOS; SBO, soybean oil; SBOMOS, soybean oil and MOS.

The principal component analysis (PCA) attributed 62.9% of the total variability on the posterior GALT-immune response in terms of relative gene expression to the dietary treatment (Figure 4). The first principal component (PC1) showed the influence of the dietary treatment on posterior gut gene expression and accounted for 41.84% of the total variability, PC1 axis separating FO-based diets from SBO diets. PC2 showed the influence of SBO or MOS addition to a FO diet without prebiotic on posterior gut relative gene expression and accounted for 21.08% of the total variability (Figure 4). Similarly the RDA indicated that the dietary treatment explains 47.64% of the total variability observed on posterior gut gene expression (*P* < 0.005). The first component (RDA1) explains 79.1% of the variability and cluster of all the genes, with exception of MHCII, on FO-based diets hemisphere. The rest of the variability (20.9%) is explained by the second component (RDA2) and clusters almost all genes related to cellular components on the axis upper space, where SBO and/or MOS where supplemented and the humoral genes on the lower axis space in relation to the absence of dietary SBO or MOS (Figure 5). This indicates that (I) SBO dietary inclusion seems to exert a stronger effect than dietary MOS when supplemented in combination, (II) fish fed FO-based diets react better to MOS supplementation than fish fed SBO-based diets, (III) fish gene expression up-regulation after dietary MOS for 60 days seems to be centralized in cellular components, and (IV) SBO supplementation caused an up-regulation of MHCII gene expression regardless of dietary MOS inclusion.

## Discussion

### Growth performance

Growth of European sea bass was not significantly affected by the complete replacement of FO by SBO in the present study, however, the FO provided by the dietary fish meal was in agreement with previous studies in the same species where up to 80% replacement of FO by SBO fed for 63 days did not significantly affected growth of this species (59, 60). However, feeding 80% SBO diet for 280 days significantly reduced SGRs (26, 60), in relation to the low contents on dietary LC-PUFA, which are known to be essential for growth (61). Similarly, in the present study there was a tendency in the SBO fed fish to have lower SGR, with fish fed SBO diet showing the lowest SGR. On the contrary, and in agreement with previous studies in the same fish species (44), MOS supplementation favored European sea bass SGR. Authors in ref. (44) related the dietary MOS positive effects on fish growth performance to (a) variations on absorption/incorporation rates of specific nutrients due to changes on intestinal pH; (b) a better functionality of the enterocyte membrane in terms of integrity and transcellular and paracellular transport rates due to changes in mucus proportions and composition; or (c) changes on the levels of peptides that control either the peripheral satiation system and the long-term system (51, 62) and provide information for the hypothalamic central feeding system that controls feed intake. Moreover, the SGR of fish fed SBO with MOS supplementation tended to be similar to that of European sea bass fed FO, thus potentially neutralizing the negative effect of SBO. However, the long-term effects of the complete FO replacement by SBO in diets supplemented with MOS should be further studied to determine the optimal MOS dose and time of supplementation in relation to fish age (44).

### Posterior gut lipid composition and fatty acid profiles

Neither dietary oil source nor prebiotic supplementation affected intestine lipid content, in agreement with previous studies (32, 63). Indeed, posterior intestine fatty acid profiles mainly reflected diet composition. However, it is remarkable that higher tissue saturated, n–3 and n–3LC-PUFA fractions in relation to their particular dietary content for fish fed SBO-based diets compared to fish fed FO-based diets, was in agreement with previous studies (32, 60), denoting the importance of these groups of fatty acids for the correct functioning of intestinal cells. Particularly, the increased deposition of total n–3 and n–3LC-PUFA, mainly as a consequence of a higher DHA and EPA tissue content, could be related to the preferential esterification of these fatty acids into intestinal polar lipids on the anterior gut independently of their dietary content as described before for this and other marine fish species (32, 60). In addition, preferential assimilation of LC-PUFAs in relation to a higher affinity of intestinal lipases and fatty acid-binding proteins (32, 64, 65) would also contribute to the high n–3LC-PUFA content in intestinal tissue. Besides, the almost comparable saturated percentages in posterior gut of fish fed SBO- and FO-based diets, despite a different dietary intake, denote a high production of these fatty acids in the liver in relation to their importance as constituents of membrane polar lipids, particularly of the phosphatidylcholine fraction (32, 66, 67). In this sense, previous studies have demonstrated that MOS supplementation increases the polar lipid fraction in European sea bass posterior gut, predominantly of the first resultant compounds of phospholipids synthesis (phosphatidylcholine and phosphatidylethanolamine), but also phosphatidylserine and phosphatidylinositol, in relation to a better intestinal mucosal integrity and to a higher production of prostaglandins levels after stimulation (63). Indeed, the slightly lower content of posterior gut ARA in fish fed MOS, regardless of dietary levels, could be related to an enhanced prostaglandins production as consequence of a higher number of infiltrated leukocytes in this tissue as previously demonstrated for the same fish species (63).

### Gut morphology and mucous cell dynamics

Regional differences in form and function of the gut are found in response to basic diet but differential effects are found due to MOS supplementation. In the anterior gut, where most enterocytes are absorptive (68), mucous cell densities are significantly higher in European sea bass given the FO diet than the SBO diet, thus supporting a possible higher mucous secretion. The density of mucous cells in the posterior gut epithelia was significantly higher relative to the anterior gut in all treatments, supporting many studies showing more mucus cells in the hindgut relative to the foregut (69). However, within the posterior intestine, the mucosal epithelium reacts to the dietary presence of SBO and MOS by decreasing the mucous cell size when MOS was combined with FO and increasing it when combined with SBO, however, this results in similar E:M ratios and thus modulates the mucosal dynamics. A similar regional disparity has been found in rainbow trout (*Oncorhynchus mykiss)* immune response to a challenge with *Aeromonas salmonicida*, where the foregut up-regulated IL-1β, TNF-α, interferon INFγ, IL-8, and TGFβ gene expression, whereas the hindgut only down-regulated its baseline expression of TGFβ (70). This differential picture of the action of mucous cells and epithelial cells suggests that far more is being activated in both gut segments and that mucosal mapping synthesizes the effects of many more genes than those which are typically targeted.

In the skin, mucous cell areas, densities and E:M were not significantly different between treatments although there was a tendency to smaller cells at a higher E:M ratio in the FO group (not significant). The mean mucous cell areas of dorsolateral skin in European sea bass ranged from about 150 to 200 μm^2^, similar to Atlantic salmon (*Salmo salar*) with a mean of about 160 μm^2^ (52). However, densities in the dorsolateral of European sea bass are generally below 5% of the epithelium, which is lower than the 7–10% found in the Atlantic salmon dorsolateral but about equal to the low density found on the salmon head and dorsal caudal peduncle (52). This method also allows distinction of regions with differing mucous cell characteristics, as found across the salmonid body (52, 71, 72) or in response to antiparasitic treatments (73). Patterns of mucous cell distribution may be correlated with differential gene expression and be a vital clue to innate immune responses to a variety of pathogens and inflammatory agents.

The index E:M showed significantly more epithelium per mucous cell (E:M) and significantly larger mucous cell areas in the skin than in both gut segments that were not affected by diet. However, the density of skin mucous cells was equal to the anterior gut and significantly lower than that of the posterior guts, and not affected by MOS or oil source. This is in accordance with previous studies in the same species fed FOMOS diets where intestinal mucus presented enhanced immune activity in terms of lysozyme and bactericidal activities when compared to fish fed FO diets, but this effect was not detected in skin mucus (44). This suggests that, in the unchallenged skin, the diet composition posed no inherent immune challenge to skin mucosa and did not stimulate differential innate immune responses, in contrast to parasitic challenges, which do induce changes in skin mucous cell dynamics in salmon (Pittman and Campo, personal communication). However, despite similar structures between mammalian and teleost mucosal epithelia in the gut and skin, teleost skin has living cells on the surface as opposed to the keratinized mammalian skin (74). As such the pathogen-associated molecular patterns (PAMPs) can be interpreted and transmitted rapidly by the living teleostean epithelial cells, giving changed skin mucus properties change in response to bacterial load in the water, as is found in carp (74, 75). However, some salmonids (*Salmo salar* and *Salmo trutta*) exhibit a whole-body mucus immune response (skin, gills) to Amoebic Gill Disease, while rainbow trout (*Oncorhynchus* mykiss) has only a local response in the gills (76). Therefore, we cannot exclude the possibility of a latent differential skin response to immune challenges in European sea bass fed different diets.

### Immune genes expression

Fish gut epithelium acts and reacts as the first line of protection against any potentially harmful substances/pathogens, dealing continuously with the resident microbiota, fish external environment, and substances/molecules coming from feeding. The results of the present study denoted a clear difference in the expression of GALT-related genes in the posterior gut of European sea bass fed for 60 days with diets containing either FO or SBO, denoting the importance of the dietary fatty acid profile on the immune system of marine fish, in agreement with previous studies (6, 77). Whereas MOS dietary supplementation in the FO-based diet exerted a clear up-regulation of genes related to GALT-cellular components, MOS addition to SBO-based diets had a milder effect. These results suggest that under a SBO-based diet, the fish GALT system is rendered less capable of mounting a response to MOS due to a lower substrate availability, an effect that is more consistent with the presence of SBO than with the presence of MOS. Nevertheless, the different pattern of GALT-related genes expression found for MOS and SBO containing diets in comparison to FO-based diets would be related to a response to non-self substances or to potential changes in the microflora caused by dietary supplementation with SBO (39) or MOS (78–80). However, both GALT-immune-related genes responses differed between SBO and MOS.

Mannan oligosaccharides supplementation, particularly in FO-based diets, triggered a posterior gut response by increasing posterior gut TNFα, CD4 and CD8 gene expression, denoting a T-cell-mediated inflammation. Interestingly, a concentrated product of MOS (cMOS; second-generation product) did not affect the expression of these genes (81), this is probably related to the lack of some residual components of the outer cell wall of *Saccharomyces cerevisiae* lost during the production process, such as β-glucans. TNFα is a proinflammatory cytokine released by macrophages or activated T-cells in response to PAMPs that play a key role as a chemotactic agent leading to the migration of leukocytes to the inflammation site (82, 83). Leukocyte migration is promoted in the posterior gut of European sea bass after MOS supplementation as described in previous studies by electron and optical microscopy [for review see Ref. (44)]. Studies on leukocytes in higher vertebrates attributed the stimulatory effect of yeast cell wall components on TNFα production/expression to NF-Kβ activation (83, 84) probably triggered by dectin-1 expression (85–88). However, in the present study, activation through mannose receptor (MR) or DC-SING and/or a modulation of the TLR3 and TLR4 should not be discarded (88–91). Besides, exposing the fish to an external agent like MOS over a relatively long (2 months) period in terms of immediate GALT response may have affected the pleiotropic role of TNFα (proinflammatory and immunosuppressive effects) and could influence the regulation of IL-6 production by antigen-presenting cells (APCs) (92). This could have contributed to attaining a possible state of homeostasis by the end of the experiment. Indeed, a higher level of TNFα transcripts in the absence of IL-10 response is normally associated with increased mucosal IL-17 in humans (93, 94) which together with IL-22 induces the production of bactericidal and bacteriostatic peptides by enterocytes (94, 95) and promotes gut integrity via increasing barrier forming claudin proteins and wound healing (95–98) as previously described for fish fed MOS during a similar period of time (44).

Mannan oligosaccharides supplementation also up-regulated CD4 and CD8 gene expression, suggesting the promotion of T-cell-specific responses by yeast cell wall components via these and other cytokines, as INFγ (84). These elevated transcripts levels could be the consequence of variations in the microbiota populations as previously described for fish fed MOS (78–80), since T-cells are extremely important in creating tolerance or immunity against commensal microbiota. In the present study, dietary MOS in FO-based diets could also promote cytotoxic T-cell immune response by up-regulating MHCI gene expression, much like in porcine bronchoalveolar lavage fluid cells (99), highlighting the role not only of APCs but also of any nucleated cell on MOS-derived GALT-related immune genes response. Contrary to this, SBO strongly up-regulated the expression of MHCII gene relative to FO, indicating possible alterations on leukocytes populations other than T-cells, such as macrophages or granulocytes, as previously reported for the same fish species (100). This could be induced by changes in the microbiota composition and/or on the short-chain fatty acids (SCFAs), such as butyrate, acetate, formate, or propionate, which can either induce mucin synthesis and mucous production or inhibit mucin synthesis *in vitro*, supporting their role in regulating mucous cell differentiation (101). These SCFAs are also end-metabolites produced by the microbiota and profoundly influence gut barrier function, as well as host immunity and cell proliferation (102). Indeed, previous studies in salmon gut epithelium fed SBM demonstrated that MHCII molecules have a polyclonal role on the gut epithelium endocytic and/or exocytic uptake, processing and presentation of luminal agents associated with maintaining intestinal homeostasis (103).

Soybean oil inclusion down-regulated COX2 expression in posterior gut of European sea bass, in relation to the alterations on the substrate EPA and ARA membrane contents in this tissue as well as on the EPA/ARA ratio. Inhibition of COX2 activity by dietary lipids has been related to a lower production of prostaglandin E2 (PGE_2_) and leukotriene B4 (LTB_4_) in fish fed SBO-based diets (10, 104–107). On the contrary, MOS inclusion increased COX2 transcripts. Indeed, the slightly lower content of tissue ARA in European sea bass fed MOS could be related to an enhanced PGs production as a consequence of a higher number of infiltrated leukocytes as previously described for the same species fed FO-based diets with MOS supplementation (62). Furthermore, PGs affect transcription of several genes involved in T-cell activation, physicochemical properties, and discharging rates of intestinal mucus and produce the closure of tight junctions via restoring occludin and ZO-1 through phosphatidylinositol-3-kinase pathway (62) as a mechanism of repairing epithelial barrier after external aggression in agreement with an improved conserved cytoarchitecture of the enterocyte in fish fed FOMOS [for review see Ref. (44)].

Activated T-cells levels may also trigger B cell activation and Ig production. Thus, in agreement with the COX2 down-regulation and potentially impaired T-cell activation, IgM chain gene expression was also down-regulated by dietary SBO inclusion. Contrary to this, the increased expression of IgM chain in posterior gut of fish fed FO-based diets with MOS points to a higher production of Ig in this intestinal section (81), and a higher plasmatic cell density compared to that of fish fed an FO diet (80). However, the effect of SBO over the B cell population in terms of IgM relative gene expression seems to be stronger than the one caused by MOS itself and points again to fish system incapability of responding properly to the MOS stimulus in fish fed SBO.

Regarding GALT humoral components, feeding SBO leads to a down-regulation of IL-1β expression, in the posterior intestine of European sea bass. On the contrary, expression of these genes in whole intestine of *Solea senegalensis* was not affected by dietary inclusion of SBO (77), what could be related to the different species, experimental conditions, or intestinal section evaluated. Indeed, among the different sections of the intestinal tract GALT activity and microbiota profiles have been found to differ (44) as well as the morphological alterations caused by SBO (29, 32, 34). MOS supplementation to SBO diets only mildly up-regulated the IL-1β gene expression in posterior gut equaling that found in FO fed fish, and, in agreement with previous studies (81), did not affect the expression of this gene when it was supplemented to FO diets. Studies in higher vertebrates peripheral mononuclear blood cells indicate that dietary MOS inclusion, rather than IL-1β, affects IL-1α (99), which stimulates growth and differentiation factors that trigger cellular proliferation and migration events in response to immunological insults (99).

Fish oil substitution by SBO also down-regulated IL-10 relative expression, in agreement with the morphometric decrease of the mucous barrier in this fish by decreasing the fold length. It is demonstrated that IL-10 preserves the mucous barrier and maintains mucin production by not exerting a stress effect on the endoplasmic reticulum of the mucous cells (108). MOS inclusion in SBO diets markedly up-regulated IL-10, denoting its important role for mucous barrier strength, increasing the mucous cell area and restoring the normal fold length in this fish. Similarly, dietary SBO down-regulated gene expression of TGFβ, whereas MOS inclusion in SBO diets up-regulated the expression of this gene. TGFβ plays a crucial role in maintaining immune homeostasis and preventing mucosal inflammation via regulating lymphocytes and phagocytes activation (109, 110). Therefore, MOS induced a homeostatic balance in the posterior gut mucosal tissue of fish fed SBO almost to the same level as with FO diets.

### General overview

After the use of FO and FM alone in the feed for European sea bass, there are signals for high cell proliferation and proinflammatory agents and cell transforming factors, which also include apoptosis. This indicates that the FO diet induced, in European sea bass intestine, a good epithelial turnover combined with differentiation of cells and that the high TGFβ perhaps directs any functional trans-differentiation of the epithelial to the mucous cells. On the other hand, in the SBO group, the entire architecture of the gut was affected, beyond the region of the lumen, inducing shorter intestinal folds and lower mucous cell density in the anterior gut than in the FO-based diets, but with average mucous cell sizes and relative amounts of epithelium to mucous cell. However, the posterior gut has only average values for the mucous cell dynamics suggesting a possible immune function of the anterior gut modulating the immune response of the next gut segment. Additionally, there is a down-regulation of immune genes like receptors on T-cells and those agents mediating inflammation. There is more MCHII to interact with cytokines but conversely there are decreases in most cytokines as well as in agents for cell proliferation, pro- and anti-inflammatories, cell death and differentiation, and fewer innate Igs. The overall view is that SBO influences processes such as fundamental cell cycling and determination and reduces the fish’s capacity to mount an immune response.

The addition of MOS to FO-based diet (FOMOS) resulted in more tightly packed mucous cells than in SBOMOS (less epithelium to mucous cell). There was also more surface-to-volume because of the small size of mucous cells allowing more efficient diffusion of immune contents in the posterior gut. Indeed, the overall results of gene expression point to a T-cell-mediated inflammation in fish fed FOMOS. This diet induces the highest reactivity to inflammation and the most Igs, but has average cell migration and proliferation. The results indicate a strengthening of the reactivity of the innate immune system without increasing or changing cell number, and a slightly increased ability for such immune substances to diffuse from a mucous cell in the posterior gut. Indeed, the addition of MOS to the SBO diet tended to neutralize the short folds induced by SBO alone, suggesting that one action of MOS is on substances impacting cell determination at levels below the IECs and probably in the lamina propria and the submucosa. The anterior mucous cells have an enhanced ability to diffuse immune molecules (smaller mucous cells). Relative to the FOMOS group, the SBOMOS diet in the anterior gut may induce some hyperplasia of the epithelium or a reduction in the transdifferentiating capacity of the epithelial cells to make mucous cells (the interpretation can go both ways). The posterior gut shows greater storage of mucous cell contents and possible dysregulation of the gut, but no hyperplasia of the epithelium or, alternately, no dysregulation of the trans-differentiation capacity of epithelial cells to mucous cells. Relative to the SBO, this MOS-supplemented diet gives the lowest cell migration, concurring with the mucous cell configurations, but enhanced cell proliferation which possibly favors the hypothesis of epithelial hyperplasia, along with generally more cell differentiation and turnover, and more anti-inflammatories. However, the overall profile of the gastrointestinal tract given SBO-based diets has not attained the level maintained by a pure fish diet.

Thus, the addition of MOS neutralized the short folds induced by SBO, suggesting that MOS acts on the SBO substances at the level of mucous cell differentiation in the epithelial cells but also at levels below the IECs and probably in the lamina propria and the submucosa. Authors in reference (44) connected the dietary MOS positive effects on fish growth performance to a better functionality of the enterocyte membrane in terms of integrity and transcellular and paracellular transport rates due to changes in mucus proportions and composition. In this study, when MOS is combined with SBO the effect on SGR is weakened, supporting the hypothesis of functionality of the membrane, but further indicating that the primary action of MOS when given at this dose is to neutralize the dysregulation induced by SBO, rather than enhancing other immune functions. A dysregulation of the tight control exerted on innate immunity and tissue integrity in the intestine can also lead to tissue irritation and/or inflammation. The microbiota supported by our differing diets has not been analyzed but their action on the genetic and mucosal results suggests that their composition may influence antigen signaling (45).

### General conclusion

The results of this study have shown that complete replacement of FO by SBO, in addition to the FO provided by the dietary fish meal itself, markedly affects the morphology and physiology of the European sea bass gut, altering the fatty acid profile of gut, down-regulating genes related to both humoral and cellular GALT in the posterior gut, reducing mucous cell density in the anterior gut, and reducing fish growth. Inclusion of MOS in diets with complete replacement of FO by SBO partially compensated for the negative effects of the VO by increasing the mucous cell density and the mucous cell area in the posterior gut and partially compensating the down-regulation caused by SBO in the expression of certain genes such as IL-6, IL-10, and TGFβ, which are related to the functioning of the mucous barrier and maintain immune homeostasis. Thus, MOS was found to be a homeostatic balancer of the mucosal barrier in the posterior gut. However, MOS inclusion in SBO diets was not able to counteract the up-regulation of MHCII or down-regulation of COX2, suggesting that with a SBO-based diet, the fish immune system is rendered incapable of mounting a complete GALT-immune response against MOS. Patterns of mucous cell distribution may be correlated with differential gene expression and be a vital clue to innate immune responses to a variety of pathogens and inflammatory agents. Nevertheless, the observed effects of both dietary oils and MOS on European sea bass gut must be the results of species-, gut region-, diet-, and immune stimulant-specific interactions within the gut responses, as well as differential actions on microbial composition. Further research in the tight junctions between intestinal epithelial cells and MOS might illustrate the mechanisms of recovering fold length and therefore intestinal barrier.

## Conflict of Interest Statement

The authors declare that the research was conducted in the absence of any commercial or financial relationships that could be construed as a potential conflict of interest.

## References

[1] SOFIA. The State of World Fisheries and Aquaculture 2014. Rome: FAO, Fisheries and Aquaculture Department, Food and Agriculture Organization of the United Nations (2014). Available from: http://www.fao.org/3/a-i3720e.pdf

[2] Ghioni C Tocher DR Bell MV Dick JR Sargent JR. Low C-18 to C-20 fatty acid elongase activity and limited conversion of stearidonic acid, 18:4 (n-3), to eicosapentaenoic acid, 20:5(n-3), in a cell line from the turbot, *Scophthalmus maximus*. Biochim Biophys Acta (1999) 1437:170–81. 10.1016/S1388-1981(99)00010-4 10064900

[3] Tocher DR Ghioni C. Fatty acid metabolism in marine fish: low activity of fatty acyl delta 5 desaturation in gilthead sea bream (*Sparus aurata*) cells. Lipids (1999) 34:433–40. 10.1007/s11745-999-0382-8 10380114

[4] Geay F Ferraresso S Zambonino-Infante JL Bargelloni L Quentel C Vandeputte M Effects of the total replacement of fish-based diet with plant-based diet on the hepatic transcriptome of two European sea bass (*Dicentrarchus labrax*) half-subfamilies showing different growth rates with the plant-based diet. BMC Genomics (2011) 12:522. 10.1186/1471-2164-12-522 22017880 PMC3377934

[5] Izquierdo MS Robaina L Juárez E Oliva R Hernández-Cruz CM Afonso JM. Regulation of growth, fatty acid composition and delta 6 desaturase expression by dietary lipids in gilthead sea bream larvae (*Sparus aurata*). Fish Physiol Biochem (2008) 34:117–27. 10.1007/s10695-007-9152-7 18649029

[6] Montero D Izquierdo MS Welfare and health of fish fed vegetable oils as alternative lipid sources to fish oil. In: Turchini G Ng W Tocher D, editors. Fish Oil Replacement and Alternative Lipid Sources in Aquaculture Feeds. Cambridge: CRC Press (2010). p. 439–85.

[7] Waagbø R Hemre J Holm CHR Lie O Tissue fatty acid composition, haematology and immunity in adult cod, *Gadusmorhua* L., fed three dietary lipid sources. J Fish Dis (1995) 18:615–22. 10.1111/j.1365-2761.1995.tb00366.x

[8] Farndale BM Bell JG Bruce MP Bromage NR Oyen F Zanuy S Dietary lipid composition affects blood leucocyte fatty acid compositions and plasma eicosanoid concentrations in European sea bass (*Dicentrarchus labrax*). Aquaculture (1999) 179:335–50. 10.1016/S0044-8486(99)00169-617466094

[9] Montero D Kalinowski T Obach A Robaina L Tort L Caballero MJ Vegetable lipid sources for gilthead seabream (*Sparus aurata*): effects on fish health. Aquaculture (2003) 225:353–70. 10.1016/S0044-8486(03)00301-620817101

[10] Mourente G Good JE Thompson KD Bell JG. Effects of partial substitution of dietary fish oil with blends of vegetable oils, on blood leucocyte fatty acid compositions, immune function and histology in European sea bass (*Dicentrarchus labrax* L.). Br J Nutr (2007) 98:770–9. 10.1017/S000711450773461X 17466094

[11] Theis F Miles EA Nebe-von-Caron G Powell JR Hurst TL Newsholme EA Influence of dietary supplementation with long-chain n-3 or n-6 polyunsaturated fatty acids on blood inflammatory cell populations and functions and on plasma soluble adhesion molecules in healthy adults. Lipids (2001) 36:1183–93. 10.1007/s11745-001-0831-4 11795850

[12] Sheldon WH Blazer VS Influence of dietary lipid and temperature on bactericidal activity of channel catfish macrophages. J Aquat Anim Health (1991) 3:87–93. 10.1577/1548-8667(1991)003<0087:IODLAT>2.3.CO;2

[13] Waagbø R Sandnes K Lie Ø Nilsen ER Health aspects of dietary lipid sources and vitamin E in Atlantic salmon (*Salmo salar*). Erythrocyte total lipid fatty acid composition, haematology and humoral immune response. Fiskeridir Skr Ser Ernaer (1993) 6:47–62.

[14] Thompson KD Henderson RJ Tatner MF A comparison of the lipid composition of peripheral blood cells and head kidney leucocytes of Atlantic salmon (*Salmo salar* L.). Comp Biochem Physiol (1995) 112B:83–92. 10.1016/0305-0491(95)00051-9

[15] Kiron V Fukuda H Takeuchi T Watanabe T Essential fatty acid nutrition and defence mechanisms in rainbow trout *Oncorhynchus mykiss*. Comp Biochem Physiol (1995) 111A:361–7. 10.1016/0300-9629(95)00042-6

[16] Bell JG Strachan F Good JE Tocher DR Effect of dietary echium oil on growth, fatty acid composition and metabolism, gill prostaglandin production and macrophage activity in Atlantic cod (*Gadus morhua* L.). Aquac Res (2006) 37:606–17. 10.1111/j.1365-2109.2006.01470.x

[17] Lin YH Shiau SY Effects of dietary blend of fish oil with corn oil on growth and non-specific immune responses of grouper *Epinephelu smalabaricus*. Aquac Nutr (2007) 13:137–44. 10.1111/j.1365-2095.2007.00458.x

[18] Montero D Grasso V Izquierdo MS Ganga R Real F Tort L Total substitution of fish oil by vegetable oils in gilthead seabream (*Sparus aurata*) diets: effects on hepatic Mx expression and some immune parameters. Fish Shellfish Immunol (2008) 24:147–55. 10.1016/j.fsi.2007.08.002 18158252

[19] Mourente G Good JE Bell JG Partial substitution of fish oil with rapeseed, linseed and olive oils in diets for European sea bass (*Dicentrarchus labrax* L.): effects on flesh fatty acid composition, plasma prostaglandins E_2_ and F_2α_, immune function and effectiveness of a fish oil finishing diet. Aquac Nutr (2005) 11:25–40. 10.1111/j.1365-2095.2004.00320.x

[20] Bell JG Tocher DR MacDonald FM Sargent JR. Effects of diets rich in linoleic (18:2n-6) and α-linolenic (18:3n-3) acids on the growth, lipid class and fatty acid compositions and eicosanoid production in juvenile turbot (*Scophthalmus maximus* L.). Fish Physiol Biochem (1994) 13:105–18. 10.1007/BF00004336 24202310

[21] Bell JG Castell JD Tocher DR MacDonald FM Sargent JR. Effects of different dietary arachidonic acid: docosahexaenoic acid ratios on phospholipid fatty acid compositions and prostaglandin production in juvenile turbot (*Scophthalmus maximus*). Fish Physiol Biochem (1995) 14:139–51. 10.1007/BF00002457 24197361

[22] Tocher DR Bell JG Farndale BM Sargent JR. Effects of dietary γ-linolenic acid-rich borage oil combined with marine fish oils on tissue phospholipid fatty acid composition and production of prostaglandins E and F of the 1-, 2- and 3-series in a marine fish deficient in δ5 fatty acyl desaturase. Prostaglandins Leukot Essent Fatty Acids (1997) 57:125–34. 10.1016/S0952-3278(97)90002-8 9250695

[23] Gjøen T Obach A Røsjø C Helland BG Rosenlund G Hvattum E Effect of dietary lipids on macrophage function, stress susceptibility and disease resistance in Atlantic salmon (*Salmo salar*). Fish Physiol Biochem (2004) 30:149–61. 10.1007/s10695-005-4318-7

[24] Ganga R Bell JG Montero D Robaina L Caballero MJ Izquierdo MS. Effect of dietary lipids on plasma fatty acid profiles and prostaglandin and leptin production in gilthead sea bream (*Sparus aurata*). Comp Biochem Physiol (2005) 142B:410–8. 10.1016/j.cbpb.2005.09.010 16257554

[25] Petropoulos IK Thompson KD Morgan A Dick JR Tocher DR Bell JG Effects of substitution of dietary fish oil with a blend of vegetable oils on liver and peripheral blood leucocyte fatty acid composition, plasma prostaglandin E_2_ and immune parameters in three strains of Atlantic salmon (*Salmosalar*). Aquac Nutr (2009) 15:596–607. 10.1111/j.1365-2095.2008.00627.x

[26] Montero D Mathlouthi F Tort L Afonso JM Torrecillas S Fernandez-Vaquero A Replacement of dietary fish oil by vegetable oils affects humoral immunity and expression of proinflammatory cytokines genes in gilthead sea bream *Sparus aurata*. Fish Shellfish Immunol (2010) 29:1073–81. 10.1016/j.fsi.2010.08.024 20817101

[27] Oxley A Jolly C Eide T Jordal AEO Svardal A Olsen RE. The combined impact of plant-derived dietary ingredients and acute stress on the intestinal arachidonic acid cascade in Atlantic salmon (*Salmo salar*). Br J Nutr (2010) 103:851–61. 10.1017/S0007114509992467 19943982

[28] Calder PC Burdge GC Fatty acids. In: Nicolaou A Kokotos G, editors. Bioactive Lipids. Bridgewater, NJ: The Oily Press (2004). p. 1–36.

[29] Olsen RE Myklebust R Kaino T Ringø E Lipid digestibility and ultrastuctural changes in the enterocytes of Arctic charr (*Salvelinus alpinus* L.) fed linseed oil and soybean lecithin. Fish Physiol Biochem (1999) 21:35–44. 10.1023/A:1007726615889

[30] Olsen RE Myklebust R Ringø E Mayhew TM The influences of dietary linseed oil and saturated fatty acids on caecal enterocytes in Arctic charr (*Salvelinus alpinus* L.): a quantitative ultrastructural study. Fish Physiol Biochem (2000) 22:207–16. 10.1023/A:1007879127182

[31] Olsen RE Dragnes BT Myklebust R Ringø E Effect of soybean oil and soybean lecithin on intestinal lipid composition and lipid droplet accumulation of rainbow trout, *Oncorhynchus mykiss* Walbaum. Fish Physiol Biochem (2003) 29:181–92. 10.1023/B:FISH.0000045708.67760.43

[32] Caballero MJ Izquierdo MS Kjorsvik E Montero D Socorro J Fernández A Morphological aspects of the intestinal cells from gilthead sea bream (*Sparus aurata*) fed diets containing different lipid sources. Aquaculture (2003) 225:325–40. 10.1016/S0044-8486(03)00299-0

[33] Caballero MJ Gallardo G Robaina L Montero D Fernández A Izquierdo MS. Vegetable lipid sources affect in vitro biosynthesis of triacylglycerols and phospholipids in the intestine of seabream (*Sparus aurata*). Br J Nutr (2006) 95:448–54. 10.1079/BJN20051529 16512929

[34] Benedito-Palos L Navarro JC Sitjá-Bobadilla A Bell JG Kaushik S Pérez-Sánchez J. High levels of vegetable oils in plant protein-rich diets fed to gilthead seabream (*Sparus aurata* L.): growth performance, muscle fatty acid profiles and histological alterations of target tissues. Br J Nutr (2008) 100:992–1003. 10.1017/S0007114508966071 18377678

[35] Leray C Chapelle S Duportail G Florentz A Changes in fluidity and 22:6n-3 content in phospholipids of trout *Salmo gairdneri* intestinal brush-border membrane as related to environmental salinity. Biochim Biophys Acta (1984) 778:233–8. 10.1016/0005-2736(84)90363-8

[36] Barton RG Cerra FB Wells CL. Effect of a diet deficient in essential fatty acids on the translocation of intestinal bacteria. JPEN J Parenter Enteral Nutr (1992) 16:122–8. 10.1177/0148607192016002122 1556805

[37] Cahu CL Infante JLZ Corraze G Coves D Dietary lipid level affects fatty acid composition and hydrolase activities of intestinal brush border membrane in seabass. Fish Physiol Biochem (2000) 23:165–72. 10.1023/A:1007807324809

[38] Jutfelt F Olsen RE Bjornsson BT Sundell K Parr–smolt transformation and dietary vegetable lipids affect intestinal nutrient uptake, barrier function and plasma cortisol levels in Atlantic salmon. Aquaculture (2007) 273:298–311. 10.1016/j.aquaculture.2007.10.012

[39] Ringø E Lodemel JB Myklebust R Jensen L Lund V Mayhew TM The effect of soybean, linseed and marine oils on aerobic gut microbiota of Artic charr *Salvelinusalpinus* L. before and after challenge with *Aeromonas salmonicida ssp. salmonicida*. Aquac Res (2002) 33:591–606. 10.1046/j.1365-2109.2002.00697.x

[40] Navarrete P Fuentes P De La Fuente L Barros L Magne F Opazo R Short-term effects of dietary soybean meal and lactic acid bacteria on the intestinal morphology and microbiota of Atlantic salmon (*Salmo salar*). Aquac Nutr (2013) 19:827–36. 10.1111/anu.12047

[41] Ouwehand AC Derrien M de Vos W Tiihonen K Rautonen N. Prebiotics and other microbial substrates for gut functionality. Curr Opin Biotechnol (2005) 16(2):212–7. 10.1016/j.copbio.2005.01.007 15831389

[42] Gaggìa F Mattarelli P Biavati B. Probiotics and prebiotics in animal feeding for safe food production. Int J Food Microbiol (2010) 141:S15–28. 10.1016/j.ijfoodmicro.2010.02.031 20382438

[43] Quigley EM. Prebiotics and probiotics; modifying and mining the microbiota. Pharmacol Res (2010) 61(3):213–8. 10.1016/j.phrs.2010.01.004 20080184

[44] Torrecillas S Montero D Izquierdo M. Improved health and growth of fish fed mannan oligosaccharides: potential mode of action. Fish Shellfish Immunol (2014) 36:525–44. 10.1016/j.fsi.2013.12.029 24412165

[45] Cario E. Microbiota and innate immunity in intestinal inflammation and neoplasia. Curr Opin Gastroenterol (2013) 29:85–91. 10.1097/MOG.0b013e32835a670e 23207600

[46] AOAC. Official Methods of Analysis of the Association of Analytical Chemistry. 15th ed Arlington, VA: AOAC (1995).

[47] Folch J Lees M Sloane-Stanley GH A simple method for the isolation and purification of total lipids from animal tissues. J Biol Chem (1957) 226:497–509. 13428781

[48] Christie WW Lipid Analysis. Second revised ed Oxford: Pergamon Press (1982). 201 p.

[49] Izquierdo MS Arakawa T Takeuchi T Haroun R Watanabe T Effect of n-3 HUFA levels in artemia on growth of larval japanese flounder (*Paralichthys olivaceous*). Aquaculture (1992) 105:73–82. 10.1016/0044-8486(92)90163-F

[50] Martoja R Martoja-Pierson M Técnicas de Histología Animal. Barcelona: Toray-Masson S.A. (1970).

[51] Torrecillas S Makol A Caballero MJ Montero D Ginés R Sweetman J Improved feed utilization, intestinal mucus production and immune parameters in sea bass (*Dicentrarchus labrax*) fed mannan oligosaccharides (MOS). Aquac Nutr (2011) 17:223–33. 10.1111/j.1365e2095.2009.00730.x

[52] Pittman K Pittman A Karlson S Cieplinska T Sourd P Redmond K Body site matters: an evaluation and application of a novel histological methodology on the quantification of mucous cells in the skin of Atlantic salmon *Salmo salar* L.”. J Fish Dis (2013) 36:115–27. 10.1111/jfd.12002 23009125

[53] Weibel E Stereological Methods. Practical Methods for Biological Morphology. London: Academic Press Inc (1979).

[54] Picchietti S Fausto AM Randelli E Carnevalli O Taddei AR Buonocore F Early treatment with *Lactobacillus delbrueckii* strain induces an increase in intestinal T-cells and granulocytes and modulates immune-related genes of larval *Dicentrarchus labrax* (L.). Fish Shellfish Immunol (2009) 26:368–76. 10.1016/j.fsi.2008.10.008 18996487

[55] Livak KJ Schmittgen TD. Analysis of relative gene expression data using real time quantitative PCR and the 2-CT method. Methods (2001) 25:402–8. 10.1006/meth.2001.1262 11846609

[56] Fowler J Cohen L Jarvis P Practical Statistics for Field Biology. NewYork, NY: Wiley (1998).

[57] Sokal RR Rolf SJ Biometry: The Principles and Practice of Statistics in Biological Research. 3rd ed New York, NY: Freeman (1995).

[58] Torrecillas S Caballero MJ Montero D Sweetman J Izquierdo MS Combined effects of dietary mannan oligosaccharides (MOS) and total fish oil substitution by soybean oil on European sea bass (*Dicentrarchus labrax*) juvenile diets. Aquac Nutr (2015). 10.1111/anu.12322

[59] Izquierdo MS Obach A Arantzamendi L Montero D Robaina L Rosenlund G Dietary lipid soruces for seabream and seabass: growth performance, tissue composition and flesh quality. Aquac Nutr (2003) 9:397–407. 10.1046/j.1365-2095.2003.00270.x

[60] Montero D Robaina L Caballero MJ Ginés R Izquiedo MS Growth, feed utilization and flesh quality of European sea bass (*Dicentrarchus labrax*) fed diets containing vegetable oils: a time-course study on the effect of a re-feeding period with a 100% fish oil diet. Aquaculture (2005) 248:121–34. 10.1016/j.aquaculture.2005.03.003

[61] Izquierdo MS Koven W Lipids. In: Holt J, editor. Larval Fish Nutrition. Oxford: Wiley-Blackwell, John Wiley and Sons Publisher (2011). p. 47–82.

[62] Terova G Forchino A Rimoldi S Brambilla F Antonini M Saroglia M. Bio-Mos: an effective inducer of dicentracin gene expression in European sea bass (*Dicentrarchus labrax*). Comp Biochem Physiol B (2009) 153:372–7. 10.1016/j.cbpb.2009.04.008 19393760

[63] Torrecillas S Makol A Betancor MB Montero D Caballero MJ Sweetman J Enhanced intestinal epithelial barrier health status on European sea bass (*Dicentrarchus labrax*) fed mannan oligosaccharides. Fish Shellfish Immunol (2013) 34:1485–95. 10.1016/j.fsi.2013.03.351 23528875

[64] Izquierdo MS Socorro J Arantzamendi L Hernández-Cruz CM Recent advances in lipid nutrition in fish larvae. Fish Physiol Biochem (2000) 22:97–107. 10.1023/A:1007810506259

[65] Mansbach CM Tso P Kuksis A Intestinal Lipid Metabolism. New York, NY: Kluwer Academic Publishing (2001). 433 p.

[66] Bell MV Henderson RJ Sargent JR. Changes in the fatty acid composition of phospholipids from turbot (*Scophthalmus maximus*) in relation to dietary polyunsaturated fatty acid deficiencies. Comp Biochem Physiol (1985) 81B:193–8. 4017539 10.1016/0305-0491(85)90182-8

[67] Mourente G Rodríguez A Tocher D Sargent JR Effects of dietary docosahexaenoic acid (DHA;22:6n-3) on lipid and fatty acid compositions and growth in gilthead seabream (*Sparus aurata* L.) larvae during first feeding. Aquaculture (1993) 112:79–98. 10.1016/0044-8486(93)90160-Z

[68] Nordrum S Bakke-McKellep AM Krogdahl A Buddington K. Effects of soybean meal and salinity on intestinal transport of nutrients in Atlantic salmon (*Salmo salar* L.) and rainbow trout (*Oncorhynchus mykiss*). Comp Biochem Physiol B Biochem Mol Biol (2000) 125:317–35. 10.1111/j.1095-8649.1974.tb04531.x 10818266

[69] Rombout HWM Abelli L Picchietti S Scapigliati G Kiron K. Teleost intestinal immunology. Fish Shellfish Immunol (2011) 31:616–26. 10.1016/j.fsi.2010.09.001 20832474

[70] Mulder IE Wadsworth S Secombes CJ. Cytokine expression in the intestine of rainbow trout (*Oncorhynchus mykiss*) during infection with *Aeromonas salmonicida*. Fish Shellfish Immunol (2007) 23:747–59. 10.1016/j.fsi.2007.02.002 17434320

[71] Pickering AD The distribution of mucous cells in the epidermis of the brown trout *Salmo trutta* (L.) and the char *Salvelinus alpinus* (L.). J Fish Biol (1974) 6:111–8. 10.1111/j.1095-8649.1974.tb04531.x

[72] Buchmann K Bresciani J. Microenvironment of *Gyrodactylus derjavini* on rainbow trout *Oncorhynchus mykiss*: association between mucous cell density in skin and site selection. Parasitol Res (1998) 84:17–24. 10.1007/s004360050350 9491421

[73] Olafsdottir SH Buchmann K. Dexamethasone treatment affects skin mucous cell density in *Gyrodactylus derjavini* infected *Salmo salar*. J Helminthol (2004) 78:87-90. 10.1079/JOH2003206 14972042

[74] Gomez D Sunyer JO Salinas I. The mucosal immune system of fish: the evolution of tolerating commensals while fighting pathogens. Fish Shellfish Immunol (2013) 35:1729–39. 10.1016/j.fsi.2013.09.032 24099804 PMC3963484

[75] Segner H Sundh H Buchmann K Douxfils J Sundell KS Mathieu C Health of farmed fish: its relation to fish welfare and its utility as welfare indicator. Fish Physiol Biochem (2012) 38:85–105. 10.1007/s10695-011-9517-9 21681416

[76] Roberts SD Powell MD. The viscosity and glycoprotein biochemistry of salmonid mucus varies with species, salinity and the presence of amoebic gill disease. J Comp Physiol B (2005) 175:1–11. 10.1007/s00360-004-0453-1 15517284

[77] Montero D Benitez-Dorta V Caballero MJ Ponce M Torrecillas S Izquierdo MS Dietary vegetable oils: effects on the expression of immune-related genes in *Senegalese sole* (*Solea senegalensis*) intestine. Fish Shellfish Immunol (2015) 44:100–8. 10.1016/j.fsi.2015.01.020 25655325

[78] Dimitroglou A Merrifield DL Moate R Davies SJ Spring P Sweetman J Dietary mannan oligosaccharide supplementation modulates intestinal microbial ecology and improves gut morphology of rainbow trout *Oncorhynchus mykiss* (Walbaum). J Anim Sci (2009) 87:3226–34. 10.2527/jas.2008-1428 19617514

[79] Dimitroglou A Merrifield DL Spring P Sweetman J Moate R Davies SJ Effects of mannan oligosaccharide (MOS) supplementation on growth performance, feed utilization, intestinal histology and gut microbiota of gilthead sea bream (*Sparus aurata*). Aquaculture (2010) 300:182–8. 10.1016/j.aquaculture.2010.01.015

[80] Torrecillas S Makol A Caballero MJ Montero D Dhanasiri AKS Sweetman J Effects on mortality and stress response in European sea bass, *Dicentrarchus labrax* (L.), fed mannan oligosaccharides (MOS) after *Vibrio anguillarum* exposure. J Fish Dis (2012) 35:591–602. 10.1111/j.1365-2761.2012.01384.x 22690841

[81] Torrecillas S Montero D Caballero MJ Robaina L Zamorano MJ Sweetman J Effects of dietary concentrated mannan oligosaccharides supplementation on growth, gut mucosal immune system and liver lipid metabolism of European sea bass (*Dicentrarchus labrax*) juveniles. Fish Shellfish Immunol (2015) 42:508–16. 10.1016/j.fsi.2014.11.033 25447638

[82] Ming WJ Bersani L Mantovani A. Tumor necrosis factor is chemotactic for monocytes and polymorphonuclear leukocytes. J Immunol (1987) 138:1469–74. 3805724

[83] Young S-H Ye J Frazer D Shi X Castranova V. Molecular mechanism of tumor necrosis factor-a production in 133-B-glucan (zymosan)-activated macrophages. J Biol Chem (2001) 276:20781–7. 10.1074/jbc.M101111200 11259437

[84] Seong SK KIM HW. Potentiation of innate immunity by β-glucans. Mycobiology (2010) 38:144–8. 10.4489/MYCO.2010.38.2.144 23956643 PMC3741566

[85] Rogers NC Slack EC Edwards AD Nolte MA Schulz O Schweighoffer E Syk-dependent cytokine induction by dectin-1 reveals a novel pattern recognition pathway for C type lectins. Immunity (2005) 22:507–17. 10.1016/j.immuni.2005.06.005 15845454

[86] Gross O Gewies A Finger K Schafer M Sparwasser T Peschel C Card9 controls a non-TLR signalling pathway for innate anti-fungal immunity. Nature (2006) 442:651–6. 10.1038/nature04926 16862125

[87] Dennehy KM Ferwerda G Faro-Trindade I Pyz E Willment JA Taylor JR Syk kinase is required for collaborative cytokine production induced through Dectin-1 and TLR. Eur J Immunol (2008) 38:500–6. 10.1002/eji.200737741 18200499 PMC2430329

[88] Huang H Ostroff GR Lee CK Wang JP Specht CA Levitz SM. Distinct patterns of dendritic cell cytokine release stimulated by fungal b-glucans and toll-like receptor agonists. Infect Immun (2009) 77:1774–81. 10.1128/IAI.00086-09 19273561 PMC2681737

[89] Ibuki M Kovacs-Nolan J Fukui K Kanatani H Mine Y. Analysis of gut immune-modulating activity of beta- 1,4-mannobiose using microarray and real-time reverse transcription polymerase chain reaction. Poult Sci (2010) 89:1894–904. 10.3382/ps.2010-00791 20709974

[90] Yitbarek A Echeverry H Bardy J Hernandez-Doria J Camelo-Jaimes G Sharif S Innate immune response to yeast-derived carbohydrates in broiler chickens fed organic diets and challenged with *Clostridium perfringens*. Poult Sci (2012) 91:1105–12. 10.3382/ps.2011-02109 22499867

[91] Brennan KM Graugnard DE Xiao R Spry ML Pierce B Lumpkins B Comparison of gene expression profiles of the jejunum of broilers supplemented with a yeast cell wall-derived mannan oligosaccharide versus bacitractin methylene disalicylate. Br Poult Sci (2013) 54:238.46. 10.1080/00071668.2013.775404 23647188

[92] Secombes CJ Wang T Bird S. The interleukins of fish. Dev Comp Immunol (2011) 35:1336–45. 10.1016/j.dci.2011.05.001 21605591

[93] Sfikakis PP. The first decade of biologic TNF antagonists in clinical practice: lessons learned, unresolved issues and future directions. Curr Dir Autoimmun (2010) 11:180–210. 10.1159/000289205 20173395

[94] Koch S Nusrat A. The life and death of epithelia during inflammation: lessons learned from the gut. Annu Rev Pathol (2012) 7:35–60. 10.1146/annurev-pathol-011811-120905 21838548

[95] Raffatellu M George MD Akiyama Y Hornsby MJ Nuccio SP Paixao TA Lipocalin-2 resistance confers an advantage to *Salmonella enterica* serotype *Typhimurium* for growth and survival in the inflamed intestine. Cell Host Microbe (2009) 5:476–86. 10.1016/j.chom.2009.03.011 19454351 PMC2768556

[96] Kinugasa T Sakaguchi T Gu X Reinecker HC. Claudins regulate the intestinal barrierin response to immune mediators. Gastroenterology (2000) 118:1001–11. 10.1016/S0016-5085(00)70351-9 10833473

[97] Brand S Beigel F Olszak T Zitzmann K Eichhorst ST Otte JM IL-28A and IL-29 mediate antipro-liferative and antiviral signals in intestinal epithelial cells and murine CMV infection increases colonic IL-28A ­expression. Am J Physiol Gastrointest Liver Physiol (2005) 289:960–8. 10.1152/ajpgi.00126.2005 16051921

[98] Pickert G Neufert C Leppkes M Zheng Y Wittkopf N Warntjen M STAT3 links IL-22 signaling in intestinal epithelial cells to mucosal wound healing. J Exp Med (2009) 206:1465–72. 10.1084/jem.20082683 19564350 PMC2715097

[99] Che TM *Effects of Mannan Oligosaccahride on Immune Function and Disease Resistance in Pigs* Ph.D. thesis, University of Illinois, Urbana-Champaign (2010)

[100] Picchietti S Guerra L Bertoni F Randelli R Belardinelli MC Buonocore AM Intestinal T cells of *Dicentrarchus labrax* (L.): gene expression and functional studies. Fish Shellfish Immunol (2011) 30:609–17. 10.1016/j.fsi.2010.12.006 21168509

[101] Wrzosek L Miquel L Noordine ML Bouet S Chevalier-Curt JM Robert V *Bacteroides thetaiotamicron* and *Faecalibacterium prausnitzii* influence the production of mucus glycans and the development of goblet cells in the colonic epithelium of a gnotobiotic model rodent. BMC Biol (2013) 11:61. 10.1186/1741-7007-11-61 23692866 PMC3673873

[102] Ashida H Ogawa M Kim M Mimuro H Sasakawa C. Bacteria and host interactions in the gut epithelial barrier. Nat Chem Biol (2012) 8:36–45. 10.1038/nchembio.741 22173358

[103] Romarheim OH Hetland DL Skrede A Øverland M Mydland LT Landsverk T. Prevention of soya-induced enteritis in Atlantic salmon (*Salmo salar*) by bacteria grown on natural gas is dose dependent and related to epithelial MHC II reactivity and CD8*a1* intraepithelial lymphocytes. Br J Nutr (2013) 10:1062–70. 10.1017/S0007114512002899 22813713

[104] Bell JG Dick JR Sargent JR. Effect of diets rich in linoleic or a-linolenic acid on phospholipid fatty acid composition and eicosanoid production in Atlantic salmon (*Salmo salar*). Lipids (1993) 28:819–26. 10.1007/BF025362361589446

[105] Bell JG Dick JR McVicar AH Sargent JR Thompson KD. Dietary sunflower, linseed and fish oils affect phospholipid fatty acid composition, development of cardiac lesions, phospholipase activity and eicosanoid production in Atlantic salmon (*Salmo salar*). Prostaglandins Leukot Essent Fatty Acids (1993) 49:665–73. 10.1016/0952-3278(93)90075-8 8248271

[106] Bell JG Farndale BM Dick JR Sargent JR. Modification of membrane fatty acid composition, eicosanoid production, and phospholipase A activity in Atlantic salmon (*Salmo salar*) gill and kidney by dietary lipid. Lipids (1996) 31:1163–71. 10.1007/BF02524291 8934449

[107] Bell JG Ashton I Secombes CJ Weitzel BR Dick JR Sargent JR. Dietary lipid affects phospholipid fatty acid compositions, eicosanoid production and immune function in Atlantic salmon (*Salmo salar*). Prostagandins Leukot Essent Fatty Acids (1996) 54:173–82. 10.1016/S0952-3278(96)90013-7 8860104

[108] Hasnain SZ Tauro S Das I Tong H Chen ACH Jeffery PL IL-10 promotes production of intestinal mucus by suppressing protein misfolding and endoplasmic reticulum stress in goblet cells. Gastroenterology (2013) 144(2):357–68. 10.1053/j.gastro.2012.10.043 23123183

[109] Strober W Fuss IJ Blumberg RS. The immunology of mucosal models of inflammation. Annu Rev Immunol (2002) 20:495–549. 10.1146/annurev.immunol.20.100301.064816 11861611

[110] Lilleeng E Penn MH Haugland O Xu C Bakke AM Krogdahl A Decreased expression of TGF-b, GILT and T-cell markers in the early stages of soybean enteropathy in Atlantic salmon (*Salmo salar* L.). Fish Shellfish Immunol (2009) 27:65. 10.1016/j.fsi.2009.04.007 19427383

